# Road transport of trapped antiprotons

**DOI:** 10.1038/s41586-026-11019-z

**Published:** 2026-09-16

**Authors:** M. Leonhardt, J. Morgner, F. Abbass, K. K. Anjum, B. Arndt, R. P. Czampiel, N. Daehnhardt, S. Endoh, T. Imamura, J. I. Jäger, L. Kürschner, B. M. Latacz, P. Micke, D. Natakala, R. Rahner, D. Schweitzer, S. Stahl, F. Völksen, H. Yildiz, K. Blaum, J. A. Devlin, Y. Matsuda, C. Ospelkaus, W. Quint, A. Soter, J. Walz, Y. Yamazaki, S. Ulmer, C. Smorra

**Affiliations:** 1 Heinrich Heine Universität Düsseldorf, Düsseldorf, Germany; 2 CERN, Geneva, Switzerland; 3 Institut für Physik, Johannes Gutenberg-Universität, Mainz, Germany; 4 GSI-Helmholtzzentrum für Schwerionenforschung GmbH, Darmstadt, Germany; 5 Max-Planck-Institut für Kernphysik, Heidelberg, Germany; 6 RIKEN, Ulmer Fundamental Symmetries Laboratory, Wako, Japan; 7 Graduate School of Arts and Sciences, University of Tokyo, Meguro, Japan; 8 Institut für Quantenoptik, Leibniz Universität, Hannover, Germany; 9 Physikalisch-Technische Bundesanstalt, Braunschweig, Germany; 10 Imperial College London, London, UK; 11 Eidgenössische Technische Hochschule Zürich, Zürich, Switzerland; 12 Helmholtz-Institut Mainz, Mainz, Germany; 13 Present Address: Friedrich-Schiller-Universität Jena, Jena, Germany

**Keywords:** Techniques and instrumentation, Exotic atoms and molecules

## Abstract

Low-energy antiprotons confined in ultrahigh-vacuum Penning traps^1^ enable precision investigations of charge, parity and time-reversal (CPT) invariance^2^ to test the fundamental symmetry between matter and antimatter. These studies are driven by the search for physics beyond the standard model of particle physics, including efforts to explain the observed cosmological matter–antimatter asymmetry. Until now, such experiments have only been possible at CERN’s Antimatter Factory^3,4^. At present, magnetic-field fluctuations caused by the facility operation limit the sensitivity of trapped-antiproton precision measurements^5^, which provide the most stringent matter–antimatter symmetry tests in the baryon sector^6–9^. This has inspired us to develop the cryogenic, open and transportable Penning-trap system BASE-STEP^5,10^, designed to relocate antiprotons into low-noise offline laboratories, a strategy expected to enable at least 100-fold improved CPT tests^9,11^. Here we demonstrate the road transport of antiprotons trapped in BASE-STEP. Ninety-two trapped antiprotons have been transported outside the Antimatter Factory along a 7.5-km route without particle loss or measurable degradation of the trap vacuum. This achievement marks the starting point for a new era of antiproton precision measurements^8,12^ in dedicated low-noise offline laboratory environments.

## Abstract (web summary)

Researchers demonstrate successful road transport of trapped antiprotons, establishing a practical method for moving antimatter to quieter laboratories and substantially improving future tests of matter–antimatter symmetry.

## Main

Experiments that compare the properties of matter–antimatter conjugates^13,14^ test the fundamental CPT invariance, which is deeply intertwined with our understanding of energy, space-time and causality^15,16^. As such, it constitutes a cornerstone of the relativistic quantum field theories of the standard model, and any observed difference would point to physics beyond it. Particularly compelling are experiments involving stable matter–antimatter systems, such as electrons and positrons^17,18^, protons and antiprotons^6–9^ or hydrogen and antihydrogen^19,20^. Stored in ultrahigh vacuum, the intrinsic stability of these systems permits non-destructive measurements^21,22^ at exceptionally long interrogation times^11^ and ultrahigh fractional precision, enabling sensitivity to potential minute signatures of exotic phenomena beyond established physics^23–28^.

In the BASE collaboration, we use an advanced cryogenic Penning-trap system to perform CPT invariance tests by high-precision comparisons of the fundamental properties of protons and antiprotons, such as charge-to-mass ratios *q*/*m* and magnetic moments^6,7^. These are extracted from single-particle measurements of the cyclotron (*ν*_c_) and spin-precession (*ν*_*L*_) frequencies, both proportional to the magnetic field *B*. Using the BASE trap system in the CERN Antimatter Factory (AMF), we have achieved relative uncertainties at the level of 16 parts per trillion for the charge-to-mass ratio^6,9^ and 1.6 parts per billion^7,8^ for the magnetic moment, constraining CPT-violating effects down to sensitivities of 2 × 10^−27^ GeV (ref. ^9^). However, these measurements are ultimately limited by fluctuations of *B* at the experiment location in the AMF, which directly contribute to the measurement uncertainty^5^. Our efforts to mitigate these limitations have included the development of the reservoir trap technique^29,30^, enabling precision measurements when the AMF is offline, typically for two to three months per year^9^. Combined with improved magnetic shielding^31^, this strategy has boosted the frequency stability and enabled our latest results^11^; further improvements toward parts-per-trillion measurements require long-term systematic studies extending well beyond annual shutdown periods and, consequently, years of data taking. Other Penning-trap systems in magnetic noise-free environments achieved *g*-factor difference measurements with 5.6 × 10^−13^ relative uncertainty^32^, high-precision magnetic moment measurements^33^ and 10^−12^ level uncertainties in *q*/*m* measurements^34,35^ on much shorter timescales. In contrast, although we demonstrated the methods required for measurements at higher precision^11,36^, progress in our experiments remains constrained by the ambient magnetic noise in the AMF.

As a key technology to overcome these limitations, we have constructed the transportable antiproton trap system BASE-STEP^5^ to decouple our measurements from the dominant source of magnetic noise. Although transporting antiprotons has been mentioned and proposed earlier in different contexts, for example in refs. ^37–39^, only the recent limits of the BASE antiproton measurements made its implementation necessary as the next logical step towards higher precision. Other projects working towards antiproton transport include PUMA^40^ and the work proposed in ref. ^41^, both exploiting antiproton annihilation for either probing nuclear skin compositions of radionuclides^38^ or producing isomeric ions for metrology applications^42^, respectively. However, only two transport studies with electrons^39^ and protons^10^ have been reported so far. These confirm the feasibility of relocating Penning-trap systems but leave unresolved whether the extreme vacuum conditions required for antiproton storage can be maintained during and after transport. Here we demonstrate the successful relocation of antiprotons using BASE-STEP^5^, transporting the device by truck across the Meyrin site of CERN.

## The BASE-STEP apparatus

**Fig. 1:**
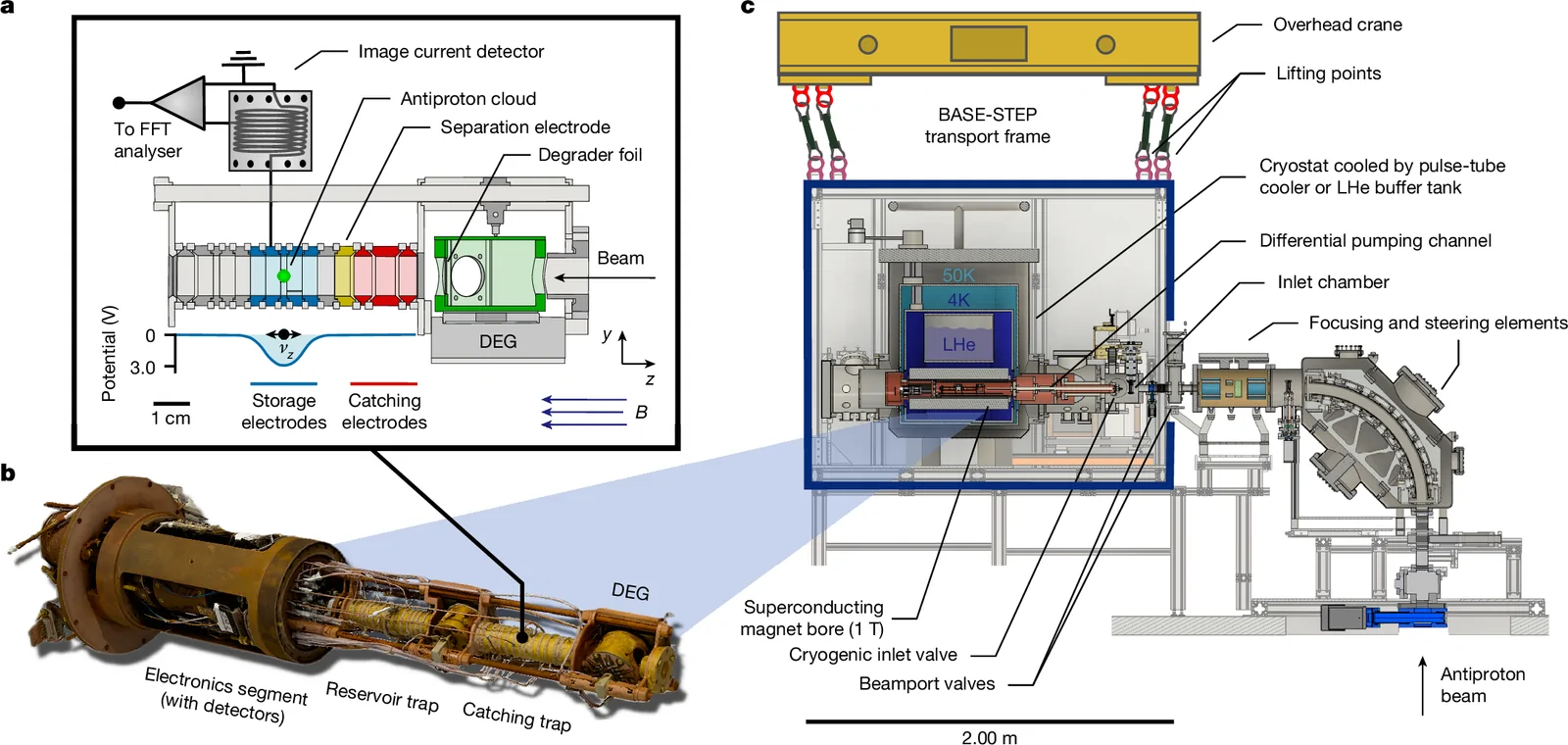
The BASE-STEP apparatus. **a**, Overview of the BASE-STEP catching trap used in this study. The positions of the high-voltage catching electrodes (red) for antiproton injection and extraction are indicated together with the storage electrodes (blue) used to prepare and monitor the antiproton reservoir. Antiprotons are injected through the rotatable DEG, confined within the central storage electrodes and non-destructively monitored using an image-current detector. **b**, Cryogenic inset of the experiment in the bore of a 1-T superconducting magnet, showing the reservoir trap, the catching trap, the DEG and the electronics segment housing the image-current detectors. **c**, Schematic of the injection set-up showing the antiproton transfer beamline and the transport frame. Antiprotons are injected with 100-keV kinetic energy guided by several steering and focusing elements, the inlet chamber and the differential pumping channel that suppresses the residual gas flow into the trap chamber. The beamline is connected to the transport frame housing the magnet cryostat through beamport valves. Closing these valves enables the BASE-STEP apparatus to be detached from the beamline and lifted out of the AMF hall by crane.

The design of the BASE-STEP apparatus (Fig. 1) and the development of the transport routine is described in refs. ^5,10^. Here we summarize the key components and procedures relevant to antiproton transport. The Penning trap confines charged particles through the superposition of a magnetic field and an electrostatic quadrupole potential^5,43,44^. In our set-up, this configuration is realized by a custom-built, transportable superconducting magnet with an adjustable magnetic field *B* of up to 1 T and a stack of cylindrical electrodes positioned within its 4 K cold bore. For this study, antiprotons are confined in the catching trap, shown schematically in Fig. 1a. The central storage electrodes form a compensated, orthogonal, open-endcap Penning trap^45^, where trapped particles oscillate harmonically along the magnetic field lines with the axial frequency set by the central ring electrode voltage *V*_R_. In the radial plane, the particles undergo a superposition of two circular motions at the modified cyclotron *ν*_+_ and magnetron *ν*_−_ frequencies, both depending in addition to *V*_R_ on *B*. Image currents induced by the axial motion enable non-destructive detection and monitoring of the trapped antiprotons^21,46,47^. The image-current detector consists of a superconducting LC circuit^22^ at a resonance frequency of 453 kHz and requires frequency matching of *ν*_*z*_ by setting *V*_R_ ≈ 3.911 V. For readout, the LC circuit is coupled to a low-noise amplifier placed in the cryogenic electronic segment interfacing the trap stack (Fig. 1b). The superconducting magnet and its cryogenic inset are operated at about 4.3 K using a hybrid cooling system based on a cryocooler and a liquid helium (LHe) reservoir (Fig. 1c). The trap system, the magnet and the beamline-to-trap-vacuum interface, which consists of the inlet chamber, a newly developed cryogenic inlet valve and the differential pumping section (see Fig. 1c and Methods for details), are enclosed in the BASE-STEP transport frame. The complete assembly has a weight of about 850 kg and can be lifted by an overhead crane.

## Antiproton injection

Antiprotons are delivered from the AD/ELENA complex of CERN^3^ and injected into the trap through electrostatic steering and focusing elements (Fig. 1c). On entering the apparatus with a kinetic energy of 100 keV, they enter through the beamline-to-trap-vacuum interface into the cryogenic trap chamber. In the subsequent degrader stage (DEG), the antiprotons propagate through a 1.8-μm-thick polyethylene terephthalate degrader foil, reducing the energy of the transmitted particles to the keV range^48,49^. Antiprotons in the low-energy tail of the resulting distribution are captured at *B* = 993 mT by injection into the three high-voltage electrodes adjacent to the DEG. Afterwards, the trapping potential is increased so that most cotrapped contaminant anions become unstable, whereas antiprotons have a higher *q*/*m* and remain confined^43^. Subsequently, the captured particles are transferred to the central region of the catching trap, and we remove cotrapped electrons and remaining negative contaminant ions including H^−^ by driving their axial or cyclotron modes and ramping to a shallow trap potential^44^.

After the trap is cleaned, its voltage is adjusted to frequency-match the axial mode of the antiprotons to the resonant frequency of the image-current detector. To this end, we cool the antiproton axial mode resistively, and a quadrupolar radio frequency drive is applied at *ν*_*z*_ + *ν*_−_ to reduce the magnetron radius and to centre the particles in the trap by means of sideband coupling^50^. This technique is called magnetron sideband cooling. Once the antiprotons reach thermal equilibrium with the detection system, their interaction produces a dip in the detector’s noise spectrum. The number *N* of trapped antiprotons can then be determined non-destructively, using the 3-dB-width Δ*ν*_*z*_ of the dip signature. For a fully thermalized ion cloud, where the trapped particles short the detection circuit noise, the dip signal-to-noise ratio equals that of the detector and was measured to be 25(2) dB. For small particle numbers, the dip width is given as^21^

1 $$\Delta {\nu }_{z}=\frac{1}{2\pi }\frac{N{q}^{2}{R}_{{\rm{p}}}}{m{D}_{z}^{2}}.$$

Here *R*_p_ ≈ 263 MΩ is the measured effective parallel resistance of the detector and *D*_*z*_ ≈ 13.3 mm is a trap-specific length. Using the catching scheme described above and detailed in Methods, we reproducibly load about 90 antiprotons per executed injection/preparation protocol.

## Antiproton transport

**Fig. 2:**
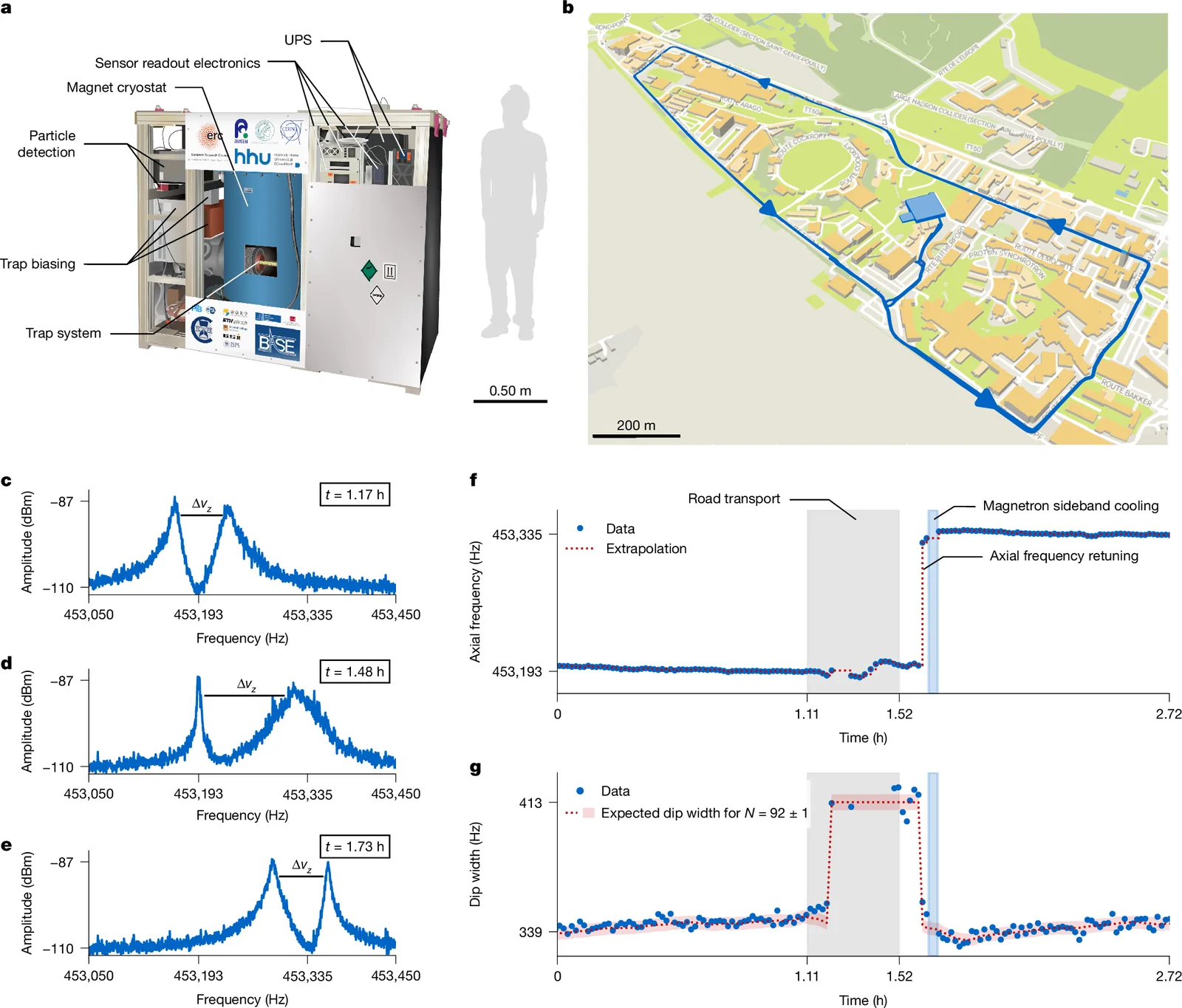
Transport and monitoring of the antiproton reservoir. **a**, Schematic of the transport frame containing the magnet cryostat with the trap system inside. The frame also houses UPS batteries, trap-biasing and amplifier power supplies, as well as particle detection and sensor readout electronics. The dimensions of the apparatus are illustrated with a person shown for scale. **b**, Road map of CERN’s Meyrin site, including GPS position data recorded during road transport. **c**–**e**, Fast Fourier Transform (FFT) spectra of the image-current signal recorded on the truck, showing the thermal noise of the superconducting LC circuit shorted by the trapped-antiproton cloud. Timestamps correspond to before the resonator shift (**c**), after the resonator shift (**d**) and after the axial frequency was retuned to the resonator (**e**). **f**,**g**, Axial frequency (**f**) and dip width (**g**) extracted from the recorded spectra. The road transport period and magnetron sideband cooling are highlighted by coloured vertical bars. The expected dip width for lossless transport, on the basis of calibration data recorded during stationary operation and including corrections for detuning-induced systematic broadening, is overlaid on the measured data. Map in **b** reproduced from OpenStreetMap under a Creative Commons licence CC BY-SA 4.0. Source data

For the transport, we loaded *N* = 92 antiprotons into the trap. After capture, the magnet is ramped down to the transport field *B* = 136 mT, decreasing the energy stored in the magnet coil. As a result, the risk and severity of magnet quenching during transport are reduced^10^. To free the transport frame for lifting, we close the two beamport valves, remove the vacuum section in between and disconnect the compressor of the cryocooler. In the configuration shown in Fig. 2a, with external cooling and power disconnected, the transport frame operates in the so-called autonomous transport mode (see ref. ^10^ and Methods for details). Uninterruptible power supply (UPS) batteries provide power for the precision voltage source to bias the trap electrodes and supply sensor readout and detector electronics, and LHe provides cooling. We have tested autonomous operation several times, reaching up to 4 h of operation without loss of the antiproton reservoir.

In autonomous mode, the apparatus was transferred by overhead crane onto a truck positioned in the AMF cargo-loading bay, using the same loading and handling procedure established for proton transport. On departure from the AMF, the onboard Global Positioning System collected real-time position data, producing the trajectory shown in Fig. 2b. The proton transport followed a shorter route, but we moved the system along an extended 3.5-km loop across the CERN Meyrin site, completing two laps in regular traffic.

Detector noise spectra of the antiproton reservoir were acquired at 1-min intervals throughout the journey (Fig. 2c–e). From these data, the axial frequency and the dip width can be extracted (Fig. 2f,g), allowing for continuous monitoring of the trapped antiprotons. During the first of the two laps, the resonator frequency shifted by 122 Hz, probably due to vibration-induced deformation of the detection wire attached to the trap, changing the circuit capacitance by about 20 fF. This resulted in a dispersive particle signal (Fig. 2d) with a systematic dip-width broadening, but left the axial frequency unaffected. Although limited data quality during transport allowed extraction of only a few dip-width points from the recorded spectra, the antiprotons were continuously tracked. We also observed a slow axial frequency drift during the entire road transport, attributed to temperature variations in the high-precision voltage source that supplies the trap electrodes and defines the axial frequency of the trapped antiprotons. In total, the apparatus travelled 7.5 km in 24 min, reaching a maximum velocity of 40.8 km h^−1^. Counting from the start, after 1.52 h, the system returned to the AMF loading bay, where the trap voltage was adjusted to retune the axial frequency to the detector (Fig. 2e), followed by magnetron sideband cooling. The apparatus was then reinstalled at the BASE-STEP beamline and reconnected to power and cooling, completing 2.72 h of autonomous operation.

During the entire transport procedure, all parameters remained within design specifications: the magnet temperature peaked at 6.2 K (below the critical temperature of greater than 8 K), and the maximum acceleration was 7.3 ms^−2^, within the 1*g* cryostat tolerance (Methods and Extended Data Fig. 7). Spectra measured over 24-h intervals before and after transport yielded dip widths of 334.08(8) and 333.92(6) Hz, respectively. Their difference was more than an order of magnitude smaller than the 3.633(16)-Hz signal expected from the loss of a single antiproton, as determined by the calibrated single-particle dip width (Methods). We therefore excluded such a loss during transport and demonstrated that transport-related vacuum effects do not induce antiproton annihilation in our system over the tested timescales.

## Storage lifetime and vacuum performance

**Fig. 3:**
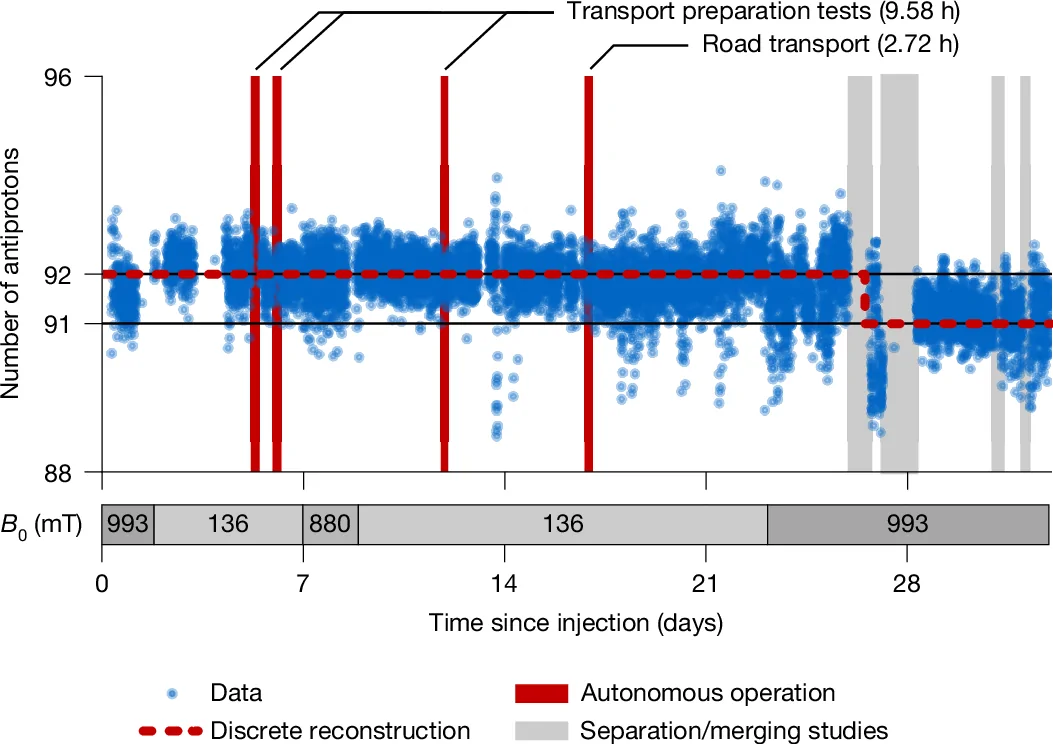
Antiproton storage for more than one month in the BASE-STEP apparatus. Measured particle number, inferred from the axial dip width, as a function of storage time since antiproton injection on 7 March 2026. Periods of particle separation and merging studies (grey) and transport preparation tests (red) are highlighted by coloured vertical bars. The transport preparation tests comprise two stationary autonomous-operation tests and one overhead crane transport before road transport. The magnetic field at the catching-trap centre is indicated along the *x* axis. The red dotted line shows the discrete antiproton number reconstruction obtained by averaging the dip-width data over 24-h intervals and rounding to the nearest integer particle number. On day 26, one antiproton was lost during separation studies (see text for details). Source data

To constrain the residual gas pressure in the catching trap and assess any effect from autonomous operation and transport, we analysed spectra collected over 33 days, extracting the number of antiprotons stored as a function of time, as shown in Fig. 3. Throughout the entire dataset, including four runs in autonomous mode totalling 12.3 h, we observed no antiproton annihilation attributable to residual gas interactions. A single-particle loss occurred 26 days after injection during trap voltage-manipulation ramps performed on the reservoir. Although hermetically sealed cryogenic systems can reach pressures below 1 × 10^−18^ mbar (refs. ^46,47^), the open catching-trap geometry for particle ejection leads to residual gas influx of helium and hydrogen. The absence of annihilation events yielded an integrated equivalent single-particle storage time of ${T}_{\bar{{\rm{p}}}}=8.28\,{\rm{a}}$. Using a Poisson process decay model and annihilation cross-sections reported earlier^46,47,51^, we obtained a lower limit on the antiproton storage lifetime in our transportable trap system of ${\tau }_{\bar{p},{\rm{lower}}}=7.27\,{\rm{a}}$ at a 68% confidence level, with corresponding upper limits on residual gas partial pressures of

2 $$\begin{array}{l}{p}_{{\rm{H}}} < 9.6\times 1{0}^{-19}\,\,{\rm{mbar}},\\ {p}_{{\rm{He}}} < 2.2\times 1{0}^{-18}\,\,{\rm{mbar}}.\end{array}$$

BASE-STEP has been designed to achieve pressures below 1 × 10^−16^ mbar to ensure antiproton storage over several months^5^; the pressure levels achieved during the present transport campaign substantially surpassed this design target.

## Transport reservoir manipulation

**Fig. 4:**
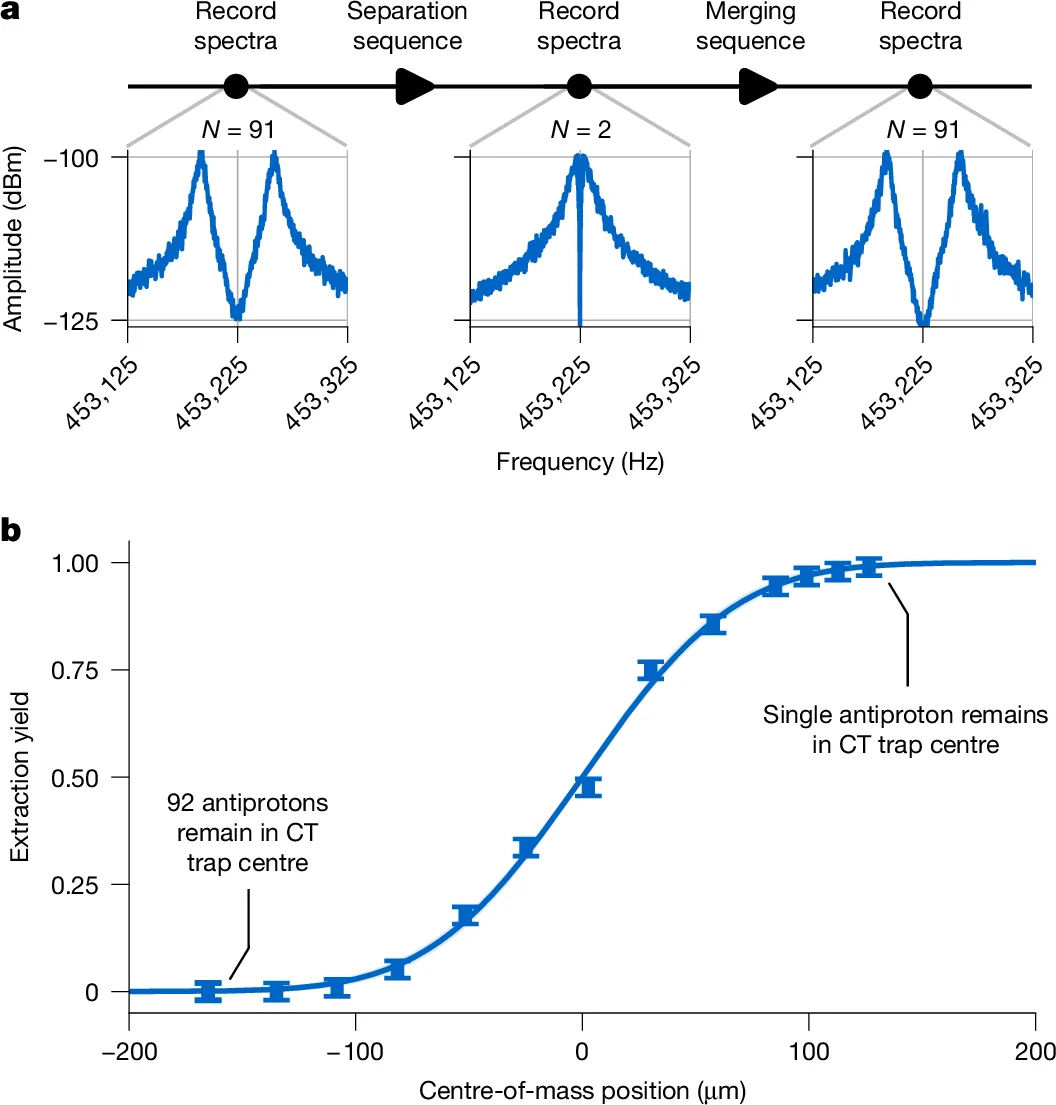
Antiproton extraction and merging. **a**, Measurement sequence used to separate antiprotons from the transported reservoir and to subsequently merge them back in. Particle numbers are tracked by means of the dip widths in the image-current detector spectra before separation, after separation and after merging. **b**, Extraction yield as a function of reservoir centre-of-mass position during separation. A yield of 1 corresponds to complete extraction to the separation electrode, and a yield of 0 indicates no extraction from the reservoir. Positive (negative) centre-of-mass positions denote displacements towards (away from) the separation electrode. The error bars represent one standard deviation (1*σ*) uncertainties. The solid line is a model fit on the basis of analytical calculations of the axial energy distribution using one-dimensional Boltzmann statistics^30^. Source data

With stable reservoir operation established, we demonstrated controlled antiproton extraction and merging, essential for offline operation. In future implementations, antiprotons will be injected from the transport trap described here into a receiver trap by means of a transfer beamline, which has been optimized by using ions produced by a separate ion source. To ensure a successful transfer, only small fractions of the reservoir, ranging from single particles to roughly 10%, will be extracted, transferred and subsequently merged in the receiver experiment to form a new reservoir. To demonstrate the required control of separation and merging, we applied the sequences sketched in Fig. 4a, adapted from the BASE trap system^30^, to the transported reservoir. During separation, carefully ramping the central ring electrode to an inverted voltage divides the cloud into two fractions. A potential offset applied to one of the correction electrodes results in an electric field that displaces the reservoir’s axial centre of mass, thereby controlling the extraction yield, defined as the ratio of separated to initial particle number. The separated fraction is transported to the separation electrode (Fig. 1a) at the end of the trap stack, and the remaining particles are retained in the trap centre. The fractions are then recombined by gradually merging the trapping potentials.

By comparing the dip widths before separation, after separation and after merging, we determine both the extraction yield as a function of centre-of-mass position (Fig. 4b) and associated losses. Magnetron heating during the voltage ramps is negligible for asymmetric splits but becomes relevant for nearly equal fractions. In those cases, we apply magnetron sideband cooling. Using this procedure, we achieved controlled separation down to a single antiproton while preserving the integrity of the reservoir, with only one antiproton lost under near-equal splitting conditions. Consequently, we can control the amount of ejected particles, which is essential to establish an antiproton reservoir that can supply offline experiments.

## Conclusion

In summary, we resolved the central, previously untested question to enable offline antiproton experiments: we showed that antiprotons can be relocated without compromising the extreme vacuum conditions required for their storage. We demonstrate a lossless transport of antiprotons across CERN in the explored regime, establishing the feasibility of offline operation with antiprotons.

Pressure constraints from more than one month of storage time remain below 2.2 × 10^−18^ mbar, to our knowledge, the lowest pressure reported for an open cryogenic Penning-trap system. This record performance establishes the BASE-STEP trap–vacuum interface as a blueprint for future offline apparatus, enabling operation over months or even years after antiproton delivery. Beyond vacuum performance, the experimental workflow has matured into a routine procedure, with repeated transport trials on the same reservoir demonstrating robust near-lossless handling. Further advances in our demonstrated methods are reliable antiproton injection and full control of separation and merge procedures down to single-antiparticle preparation.

As a next crucial step, we are planning to demonstrate the controlled antiproton transfer to receiver experiments installed for example at CERN and Heinrich Heine University in Düsseldorf, Germany. Highly efficient transfer between trap systems by means of electrostatic^52^ and magnetic beamlines^53^ has already been demonstrated and must now be implemented for our systems. To account for potential transfer losses, we intend to increase the antiproton reservoir size. We expect that antiproton clouds up to 1,000 particles can be stabilized by sideband cooling. However, large ion clouds become increasingly sensitive to torque-driven radial expansion^54^. Therefore, for the transport of larger clouds, the trap system will be upgraded with a rotating-wall drive^55,56^. Furthermore, we will proceed with the development of long-distance truck transport using a dedicated mobile power and cooling system, conceptually similar to ref. ^40^.

These advances will complete the transition to antiproton precision experiments beyond their production site, enabling at least 100-fold improvements in measurements of fundamental antiproton properties and higher-precision CPT invariance tests^7,9,11^. Finally, transportable trap technology can be extended to other charged particles, providing a foundation for high-precision measurements across a broader range of exotic ions^57,58^ and charged antimatter systems^59,60^.

## Methods

### Antiproton reservoir preparation

**Extended Data Fig. 1:**
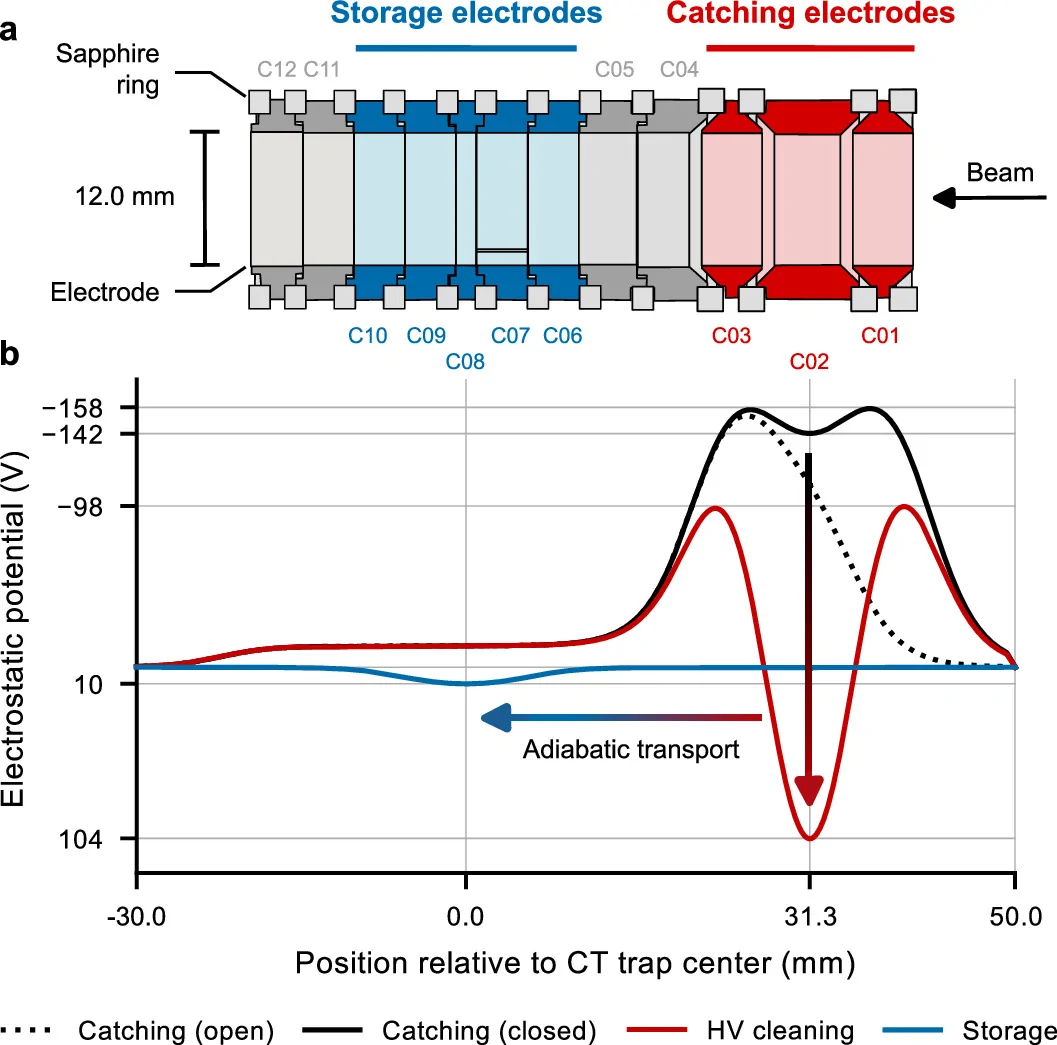
Antiproton catching potentials. **a**, Cross-sectional illustration of the BASE-STEP CT showing all electrodes biased during the catching procedure. Each electrode is labeled (C01–C12), numbered along the antiproton beam direction (C01 at entrance). The storage electrodes (blue) and the high-voltage catching electrodes (red) are highlighted. **b**, Sequence of on-axis potentials applied during the antiproton catching procedure.

The BASE-STEP catching trap is constructed of 15 cylindrical electrodes arranged in a coaxial stack (Extended Data Fig. 1a). Sapphire rings ensure electrical insulation and mechanical alignment. Antiprotons emerging from the degrader foil follow the magnetic field lines (*B* = 993 mT) into the trap, where three high-voltage electrodes (C01–C03) on the degrader side capture them. These electrodes form a nested axial potential well floated to −142 V with a depth of 16 V (Extended Data Fig. 1b). For capture, we pulse beam-side electrode C01 from 0 V to −158 V in 30 ns using a high-voltage switch. This pulse closes the potential well centred on C02 and traps antiprotons from the low-energy tail of the degraded beam.

After capture, cotrapped electrons—secondary particles forced out of the degrader—sympathetically cool the antiprotons^61^. We then ramp the C02 voltage to increase the trapping potential to 200 V, which destabilizes most contaminant negative ions while preserving antiproton confinement because of their higher *q*/*m*. We subsequently transport the particles to the central region of the catching trap. To this end, we configure electrodes C01–C03 to form a 20-V well and shift the axial potential adiabatically through the stack (typical electrode ramp time: 1 s). Finally, we confine the particles in a compensated, orthogonal five-electrode Penning trap formed by the central ring electrode C08, correction electrodes C07 and C09 and grounded endcaps starting at C06 and C10.

For non-destructive antiproton detection, we remove residual contaminants that would otherwise distort particle motion by means of space-charge effects and prevent formation of a stable axial dip signal. The dominant residual species are electrons and H^−^ ions, which cannot be eliminated by high-voltage cleaning because of their similar or higher *q*/*m*.

**Extended Data Fig. 2:**
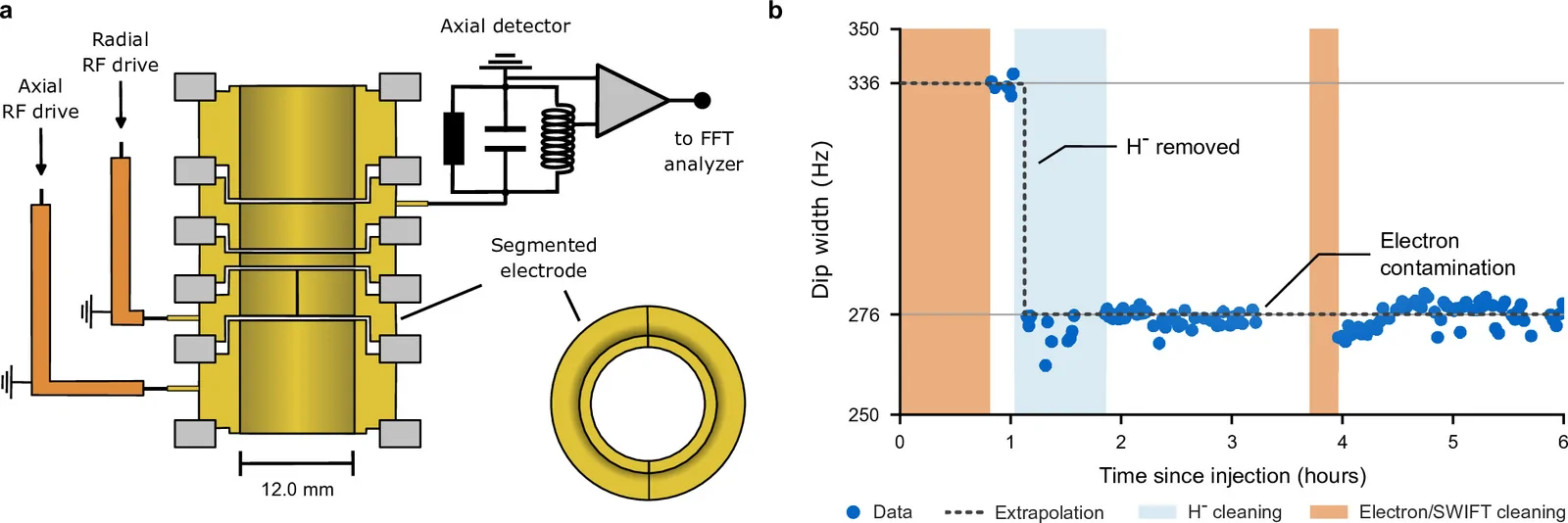
Antiproton reservoir cleaning. **a**, Shielded excitation lines deliver RF drives for axial excitation to an endcap electrode and for radial excitation to a segmented correction electrode. **b**, Co-trapped electrons and negative ions, including H^−^, are removed by resonantly exciting their axial (electron/SWIFT cleaning) or cyclotron (H^−^ cleaning) motion and ramping the trap potential to a shallow depth. Removal of H^−^ leads to a reduction in dip width (1 h after injection), whereas electron contamination perturbs the dip-width measurement and produces a gap in the recorded data (3.3 h).

We remove cotrapped electrons by applying an axial dipolar drive at 9.59 MHz to an endcap electrode (Extended Data Fig. 2a). By reducing the trap depth to 1 V, we tune the electron axial frequency into resonance with the applied drive. We then further reduce the trap depth to 0.5 V, evaporating the excited electrons. We repeat this sequence until no electrons remain. The drive is sufficiently detuned from the motional frequencies of antiprotons and H^−^ ions to avoid unintended excitation. Following injection, we repeat electron cleaning as needed to remove beta-decay-induced electron contamination from activated surfaces (Extended Data Fig. 2b). We then tune the particle cloud into resonance with the axial detector (453 kHz) and apply magnetron sideband cooling. As described in the main text, at thermal equilibrium, the particle–detector interaction produces a characteristic dip in the detector noise spectrum.

Next, we remove heavier residual ions that persist after the initial high-voltage ramp using stored waveform inverse Fourier transform excitation^62^ applied to the endcap electrode. The drive spans 20–380 kHz, exciting their axial motion. By subsequently reducing the trap depth to 0.5 V and repeating the sequence, we obtain a reservoir containing only antiprotons and H^−^ ions.

Antiprotons and H^−^ ions exhibit nearly identical axial frequencies (separated by about 250 Hz) and indistinguishable dip widths per particle, such that both species contribute to a combined dip signal. At the applied catching parameters, we typically obtain a mixed particle cloud consisting of about 80% antiprotons and 20% H^−^ ions. To discriminate between them, we exploit the difference in modified cyclotron frequency, which amounts to about 16 kHz at *B* = 993 mT. Using a single trapped proton as a magnetic field probe before injection, we determine

3 $${\nu }_{+,\bar{p}}=15.140\,{\rm{MHz}}\,,\qquad {\nu }_{+,{H}^{-}}=15.124\,{\rm{MHz}}.$$

We selectively remove H^−^ ions by applying a radial dipolar radio frequency drive to a segmented correction electrode (Extended Data Fig. 2a). A frequency sweep from 15.130 to 15.120 MHz resonantly excites the H^−^ modified cyclotron motion, increasing its radius until radial confinement is lost while leaving antiprotons unaffected. We verify successful removal by monitoring the dip width before and after excitation (Extended Data Fig. 2b). We repeat the sweep until the dip width no longer decreases (typically after one sweep; at least three are applied for robustness), yielding a clean antiproton reservoir.

Given the procedure described above, we prepare antiproton reservoirs containing about 90 antiprotons per beam injection.

### Particle-number calibration

**Extended Data Fig. 3:**
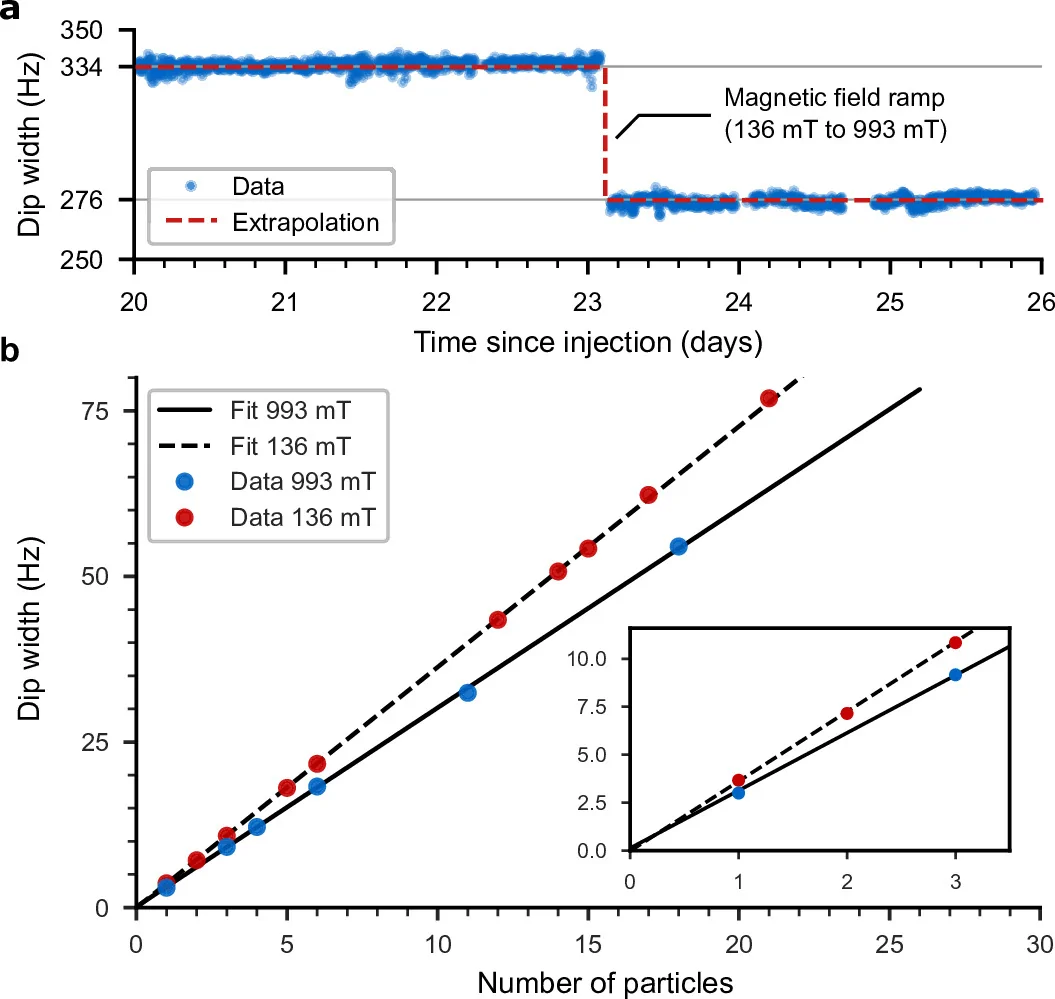
Dip width calibration. **a**, Measured dip width of the transport reservoir as a function of storage time following antiproton injection. A distinct change in dip width is observed after ramping the magnetic field (23 d after injection). **b**, Evaporation measurements used for particle-number calibration, showing dip width versus particle number. Data points correspond to measurements at 136 mT and 993 mT, and black lines indicate linear fits defining the calibration functions. The inset shows an expanded view of the low-particle-number region (*N* = 1–3), where deviations from the linear calibration model are most apparent. The statistical uncertainties of the measured dip widths are smaller than the marker size and are therefore omitted for clarity.

The particle number *N*, as shown in equation (1), is determined from the linear dependence of the axial resonator dip width Δ*ν*_*z*_ on the number of trapped particles. The relation can be simplified to

4 $$\Delta {\nu }_{z}=\Delta {\nu }_{z,1}N,$$

where Δ*ν*_*z*,1_ denotes the single-particle dip width. This proportionality enables extraction of *N* from measured spectra once a calibration of Δ*ν*_*z*,1_ has been established. Calibration measurements use proton and antiproton clouds of different sizes. We prepare clouds containing about 20 particles in the catching trap, as larger particle numbers require longer averaging times for precise counting. The clouds are then reduced stepwise to a single particle by controlled evaporation. At each step, we record the spectra and fit the dip line shape^21^ to determine Δ*ν*_*z*_. The final single-particle signal provides a reference for Δ*ν*_*z*,1_, allowing assignment of particle numbers to intermediate cloud sizes. Linear fits of Δ*ν*_*z*_(*N*) yield the calibration functions. Proton and antiproton data are treated equivalently, as their Δ*ν*_*z*,1_ values are identical. The dip width depends on the magnetic field, as the effective parallel resistance *R*_p_ of the superconducting resonator varies with field strength. Following a magnetic-field ramp from 136 to 993 mT, we observe a reduction in dip width (Extended Data Fig. 3a), consistent with a decrease in *R*_p_ at higher magnetic fields^63^. The single-particle dip width scales linearly with *R*_p_:

5 $$\Delta {\nu }_{z,1}=\frac{1}{2\pi }\frac{{q}^{2}}{m{D}_{z}^{2}}{R}_{{\rm{p}}}.$$

We therefore perform separate calibration measurements for each magnetic field. Linear fits of Δ*ν*_*z*_(*N*) for 136 and 993 mT (Extended Data Fig. 3b) yield

6 $$\begin{array}{c}\Delta {\nu }_{z}(993\,\mathrm{mT})=3.004(24)\,\mathrm{Hz}\,\cdot N\,+\,0.129(166)\,\mathrm{Hz},\\ \Delta {\nu }_{z}(136\,\mathrm{mT})=3.633(16)\,\mathrm{Hz}\,\cdot N\,-\,0.017(105)\,\mathrm{Hz},\end{array}$$

where the intercepts are consistent with zero within uncertainty. The calibration data shown were obtained from a proton cloud with *N* = 21 and an antiproton cloud with *N* = 18. The low-particle-number region is particularly important for the calibration, as uncertainties in the single-particle reference directly affect the assignment of particle numbers. The inset in Extended Data Fig. 3b highlights this region and demonstrates the agreement of the particle number assignment with the linear calibration model.

For 880 mT, where no direct calibration measurement is available, the dip width per particle is inferred from the measured detector *R*_p_. Using the proportionality Δ*ν*_*z*,1_ ∝ *R*_p_ and comparison to calibrated field settings, we obtain Δ*ν*_*z*,1_(880 mT) ≈ 3.07(4) Hz.

**Extended Data Fig. 4:**
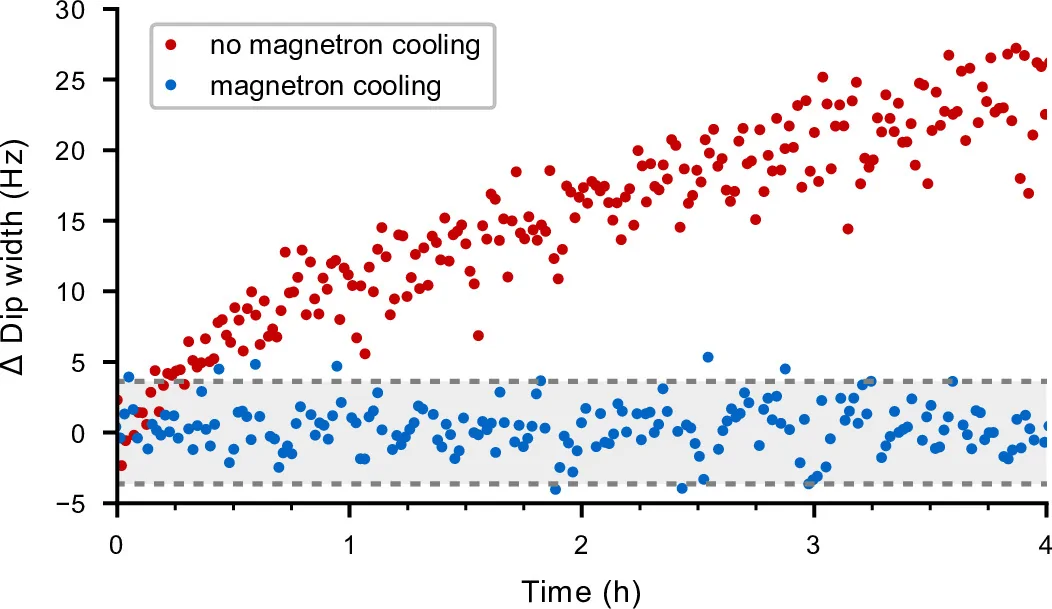
Antiproton dip stabilization. Deviation of the measured dip width from the expected value for N=92 antiprotons over two overlaid 4 h data sets, with consecutive measurements taken at 64 s averaging time. In one data set, no magnetron sideband cooling was applied, resulting in an increasing deviation over time. In the other, magnetron sideband cooling was applied for 64 s after every six measurements, keeping the dip-width deviation centered within the  ± 1 antiproton range indicated by the grey shaded area. Error bars are omitted for clarity. The measurement scatter is represented by the distribution of the data points, with detrended RMS residuals of 2.09 Hz (without magnetron cooling) and 1.68 Hz (with magnetron cooling).

To monitor the antiproton number in the transport reservoir over time, we implement an automated measurement sequence recording image-current spectra with an averaging time of 64 s. The stored antiproton cloud undergoes slow radial expansion, an effect that has been observed and studied in detail in other traps^54,64^. In general, cylinder-symmetry breaking components of the electric field apply a torque to the trapped ion cloud driving the radial expansion. Here these arise potentially from a small tilt between the electric and magnetic field axes, patch potentials or leakage currents on the segmented electrodes. The radial expansion results in a shift of the axial frequency and also in an increased dip width due to the residual potential imperfections in the trap (Extended Data Fig. 4). Compared to our proton tests, during which radial expansion has not been observable on a time scale of 4 h (ref. ^10^), the present antiproton measurements exhibit a faster radial expansion, indicating a larger torque acting on the stored cloud. The origin of this increased torque has not been identified, and the trap configuration has remained unchanged since the proton measurements. Nevertheless, the observed behaviour is consistent with a torque-driven radial expansion, as the frequency shift can be reset by applying a magnetron cooling drive^64^. Regular magnetron cooling cycles are therefore required to reset the radial expansion and enable an accurate particle number extraction.

To suppress this systematic effect, we apply magnetron sideband cooling in 6-min intervals, which keeps the cloud centred in the trap. The residual drift corresponds to a particle-number offset of only 0.1(2) antiprotons for a cloud of *N* = 92. Further systematic increases of the dip width occur if the tuning ratio and frequency detuning between particles and resonator deviate from their optimum values. However, these have been optimized so that their contribution to the particle number is not significant. For substantially larger clouds containing more than 1,000 antiprotons, however, we expect these effects to introduce significant uncertainties. In this regime, the application of a rotating-wall drive becomes desirable for reliable particle-number determination.

Over a period of 33 d, we acquired more than 26,000 spectra and extracted the dip width from each spectrum by fitting the appropriate line shape^21^. For our evaluation, we average the dip width over 24-h intervals, apply the magnetic-field-dependent calibration functions and round to the next integer number to determine the particle number. In this way, the statistical deviation from the integer number is small and systematic shifts of order 0.1 particle numbers do not affect the extracted particle number.

During the first 4 days, non-optimized trap parameters resulted in dip-width broadening, leading to a systematic overestimation of the particle number. Therefore, these data required a further correction. After determining the particle number with optimized settings, we repeated measurements at 136 and 993 mT under identical non-optimized conditions and compared them to spectra recorded with optimized settings. For these settings, we obtained correction factors of *k* = Δ*ν*_*z*,true_/Δ*ν*_*z*,measured_:

7 $$\begin{array}{l}k(993\,\,{\rm{mT}})=0.9779(11),\\ k(136\,\,{\rm{mT}})=0.99395(54).\end{array}$$

The evaluation procedure in this section yields the time evolution of the antiproton number shown in Fig. 3 of the main text.

### Storage time and trap vacuum pressure

As noted in the main text, only a single antiproton was lost over the full 33-day storage period. The loss occurred during voltage ramp sequences applied to the reservoir as part of manipulation studies. Because these ramp sequences permitted only intermittent monitoring of the reservoir, the exact time of particle loss cannot be determined precisely. On the basis of the available spectrum data, we assigned an uncertainty of 3 h to the loss time.

Using the event timeline in Extended Data Table 1, we obtained a total antiproton storage duration of 32 days, 22 h, 38 min. This corresponds to 26 days, 13 h, 01 min ± 3 h for *N* = 92 and 6 days, 9 h, 37 min ± 3 h for *N* = 91. By weighting the storage time with the particle number and summing the contributions for *N* = 92 and *N* = 91, we obtained an integrated equivalent single-particle storage time of ${T}_{\bar{{\rm{p}}}}=8.28025(34){\rm{a}}$.

We model antiproton annihilation as a Poisson process, *f*(*n*; *λ*) = *λ*^*n*^*e*^−*λ*^/*n*!, with expectation value $\lambda ={T}_{\bar{{\rm{p}}}}/{\tau }_{\bar{p},{\rm{lower}}}$. Assuming zero observed annihilation events (*n* = 0), the lower bound on the antiproton lifetime at a given confidence level (CL) is obtained from the probability of observing zero events:

8 $${\rm{CL}}=1-{\epsilon }=1-f(0;\lambda ),$$

with

9 $$f(0;\lambda )=\exp \left(-\frac{{T}_{\bar{{\rm{p}}}}}{{\tau }_{\bar{p},{\rm{lower}}}}\right).$$

This yields a lower limit on the antiproton storage lifetime under our experimental conditions of ${\tau }_{\bar{p},{\rm{lower}}}=7.27\,{\rm{a}}$ at a 68% confidence level.

The pressure limits reported in the main text are based on following approach: the conversion of the storage lifetime into an upper limit for the partial pressure of one residual gas component is performed by calculating the reaction rate on the basis of the cross-section of the annihilation reaction:

10 $$R=\frac{1}{\tau }=\frac{{v}_{{\rm{rel}}}}{\lambda }=\sigma {n}_{\mathrm{gas}}{v}_{\mathrm{rel}}$$

where *λ* is the mean free path of the particle in the residual gas, *v*_rel_ is the relative velocity between the particle and the gas molecules, *σ* is the reaction cross-section and *n*_gas_ is the density of the residual gas. This density is then converted into the pressure *p* using the ideal gas law:

11 $${p}_{\mathrm{gas}}={n}_{\mathrm{gas}}{k}_{B}{T}_{\mathrm{gas}}=\frac{{k}_{B}{T}_{\mathrm{gas}}}{\tau \sigma {v}_{\mathrm{rel}}}.$$

The temperature of the residual gas inside the trap chamber is measured by the thermometer on the magnet coil body, which has a strong thermal connection to the walls of the trap chamber and is typically at *T*_gas_ = 4.3 K. We assume here that the residual gas is composed of only hydrogen molecules and helium atoms and that all other gases freeze out in the differential pumping section or trap chamber before reaching the trap centre. Because the residual gas composition is not known, the upper limit on the total pressure in the trap chamber is set by the highest partial pressure limit and the assumption that this component makes up 100 % of the residual gas pressure.

The remaining task is now to insert the annihilation cross-section and the relative velocity. The frequently used cross-section formulas for the annihilation reaction with hydrogen^30,40,46,47^ trace back to studies about protonium formation^51,65–67^ and approximate the reaction as being with atomic hydrogen. We use the cross-section derived in ref. ^66^ as

12 $$\sigma =3\pi {a}_{0}^{2}\sqrt{{E}_{0}/E}=6\pi {a}_{0}^{2}\sqrt{\frac{{E}_{0}}{{m}_{\bar{p}}}}\frac{1}{{v}_{\mathrm{rel}}},$$

where *a*_0_ is the Bohr radius and *E*_0_ = 27.2 eV, twice the binding energy of the hydrogen atom. Notably, the 1/*v*_rel_ scaling makes the reaction rate and consequently the pressure limits independent of *v*_rel_ and therefore also of the effective noise-temperature of the detection circuit that defines the trapped particle velocity. The same applies for the annihilation cross-section of helium, where the Langevin cross-section is used^51^, so that a determination of *v*_rel_ becomes unnecessary to report the pressure limits in the main text.

We also consider a conservative scenario in which the antiproton loss is attributed to annihilation with residual gas rather than to the voltage ramps applied during separation and merging. Under this assumption, a likelihood analysis yields a lower bound on the storage lifetime of ${\tau }_{\bar{p},{\rm{lower}}}=3.6{\rm{a}}$ at the 68% confidence level, with a maximum likelihood estimate of 8.3a. The corresponding pressure constraint relaxes to an upper limit of 4.4 × 10^−18^ mbar, with a maximum likelihood value of 1.9 × 10^−18^ mbar, which remains below the upper limit quoted in the main text under the assumption of no annihilation events. Even this conservative bound of 4.4 × 10^−18^ mbar represents, to our knowledge, the lowest pressure reported for an open cryogenic Penning-trap system and exceeds the minimum operational requirements by more than an order of magnitude. However, given the temporal coincidence of the loss event with the ramp sequence, together with comparable loss behaviour observed for other antiproton clouds under similar manipulation conditions in the same apparatus, a chance annihilation event due to residual gas is highly unlikely.

### Inlet valve and vacuum interface

**Extended Data Fig. 5:**
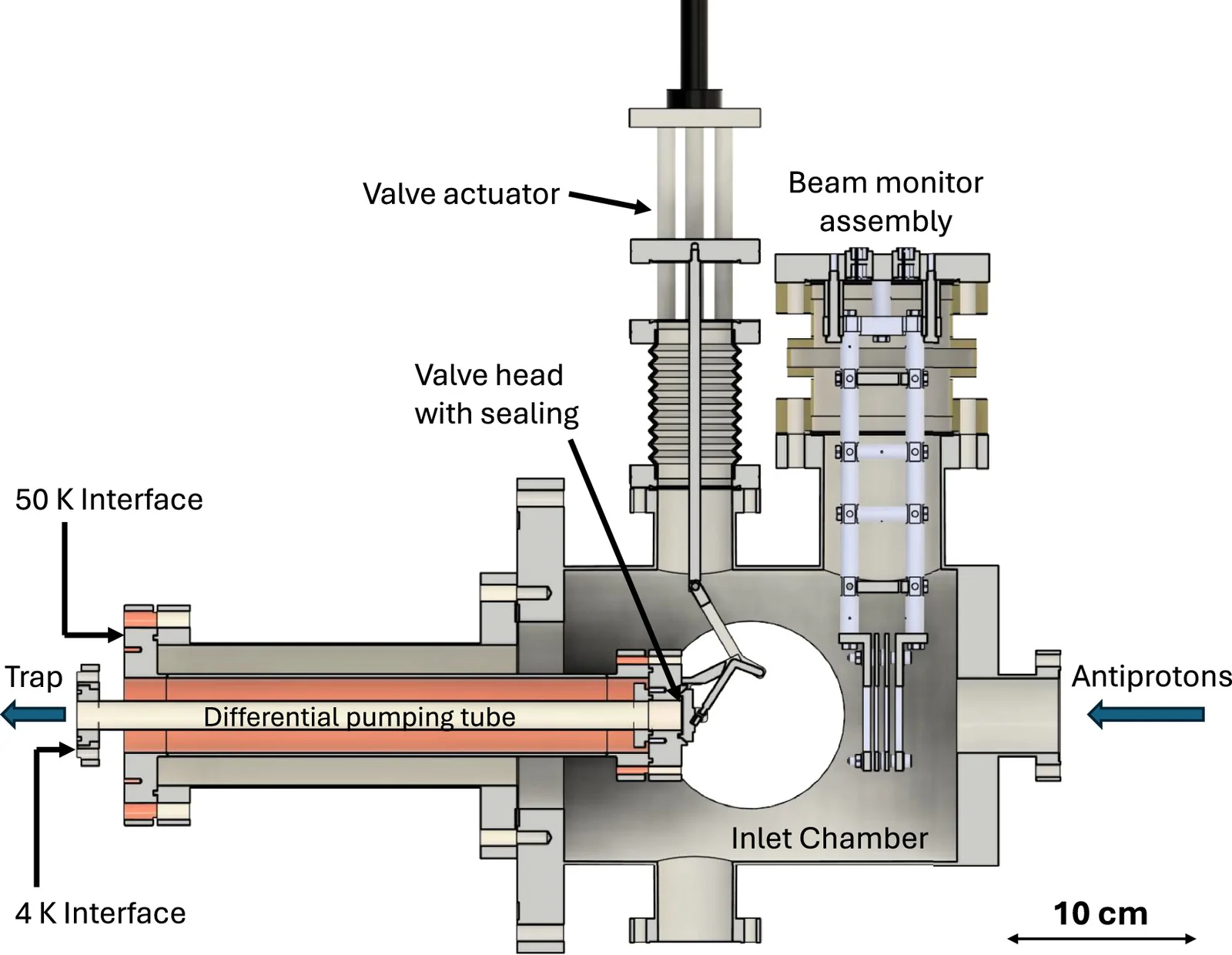
Upgraded cryogenic valve. Schematic of the vacuum interface between the beamline and the trap, showing the differential pumping channel and the inlet chamber. The cryogenic inlet valve, and the beam monitor can be moved in and out of the beam path using linear actuation stages.

The injection beamline is operated at local pressures from 6 × 10^−9^ to 3 × 10^−8^ mbar, determined by the outgassing, conductance and pumping speed along the line. The inlet-chamber pressure is held below 1 × 10^−9^ mbar using a non-evaporative getter (NEG) pump during trap operation and transport and also a turbomolecular pump while the valves to the injection beamline are open. The differential pumping section is described in ref. ^5^ and connects the vacuum of the inlet chamber and the trap chamber for the antiproton transfer with a conductance of 0.16 l s^−1^. It also manages the thermal conductance of the vacuum chamber from room temperature to 4 K by means of a set of concentric tubes. The inlet valve located at the entrance of the differential pumping channel is connected through a copper tube to the 50 K heatshield of the magnet. It is supposed to be opened only for the antiproton transfer and closed during storage and transport operations. This reduces the residual gas flow into the trap chamber and increases the monolayer formation time. The inlet valve used in refs. ^5,10^ has been exchanged for a newly developed version shown in Extended Data Fig. 5. It improves the sealing, mechanics and heat load compared to the previous version. In particular, the valve head presses on a polytetrafluoroethylene gasket when closing and is supposed to have a lower leak rate than the previously installed metal-on-metal sealed version.

### Transport set-up

**Extended Data Fig. 6:**
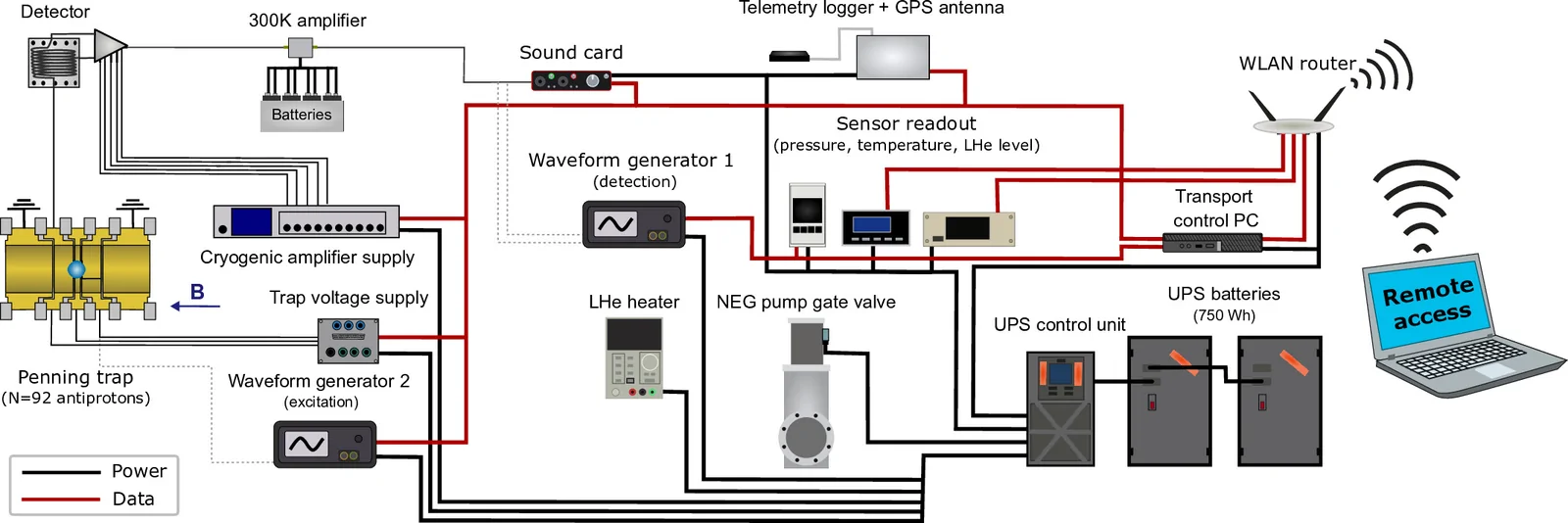
Transport mode set-up. Overview of the devices used in the autonomous transport mode and their respective power and data connections. The BASE-STEP apparatus can be remotely controlled and monitored via a laptop or smartphone.

Extended Data Fig. 6 summarizes the electronic set-up integrated in the transport frame (Fig. 2a) for autonomous operation. The antiproton cloud is confined within the central ring electrode (C08) of the catching trap. Axial confinement is provided by biasing the ring and adjacent correction electrodes with a high-precision voltage supply. All remaining electrodes are grounded through relays at the room-temperature interface.

The antiproton cloud is monitored by means of the noise spectrum of the image-current detector using a sound-card-based FFT system. The cryogenic amplifier is powered by a precision DC supply, and the room-temperature amplifiers operate from battery power. Magnetron sideband cooling is implemented through a waveform generator connected to the radial excitation line of the catching trap. The transport frame further incorporates a LHe heater element on the LHe tank to increase the gas flow for cooling of the magnet heat shields by heat transfer to the cold gas in heat-exchanger elements on the exhaust line. Before transport, the turbomolecular-pump gate valve on the inlet chamber is closed, whereas the NEG-pump valve remains open to maintain vacuum conditions in the inlet chamber. Consequently, continuous power is supplied to the NEG-pump valve to prevent automatic closure during transport.

All transport electronics except the room-temperature amplifiers are powered by a dedicated UPS; the room-temperature amplifiers are powered by their own batteries (Extended Data Fig. 6). The total transport power consumption of the UPS-supported electronics is about 170 W (Extended Data Table 2). The UPS has a capacity of about 750 Wh, providing around 4.5 h of operation after the BASE-STEP apparatus has been disconnected from external power. All voltage supplies, waveform generators, the sound card, sensor readout devices and a telemetry logger are connected to the transport control PC, a frame-mounted mini-PC. It continuously monitors the particle signal, cryostat temperatures, telemetry sensors, LHe level and vacuum pressures. The PC also controls electrode voltages and radio frequency drives for magnetron sideband cooling during transport. After disconnection from external infrastructure, remote access to the transport control PC is established by means of a wireless network.

### Further transport data

**Extended Data Fig. 7:**
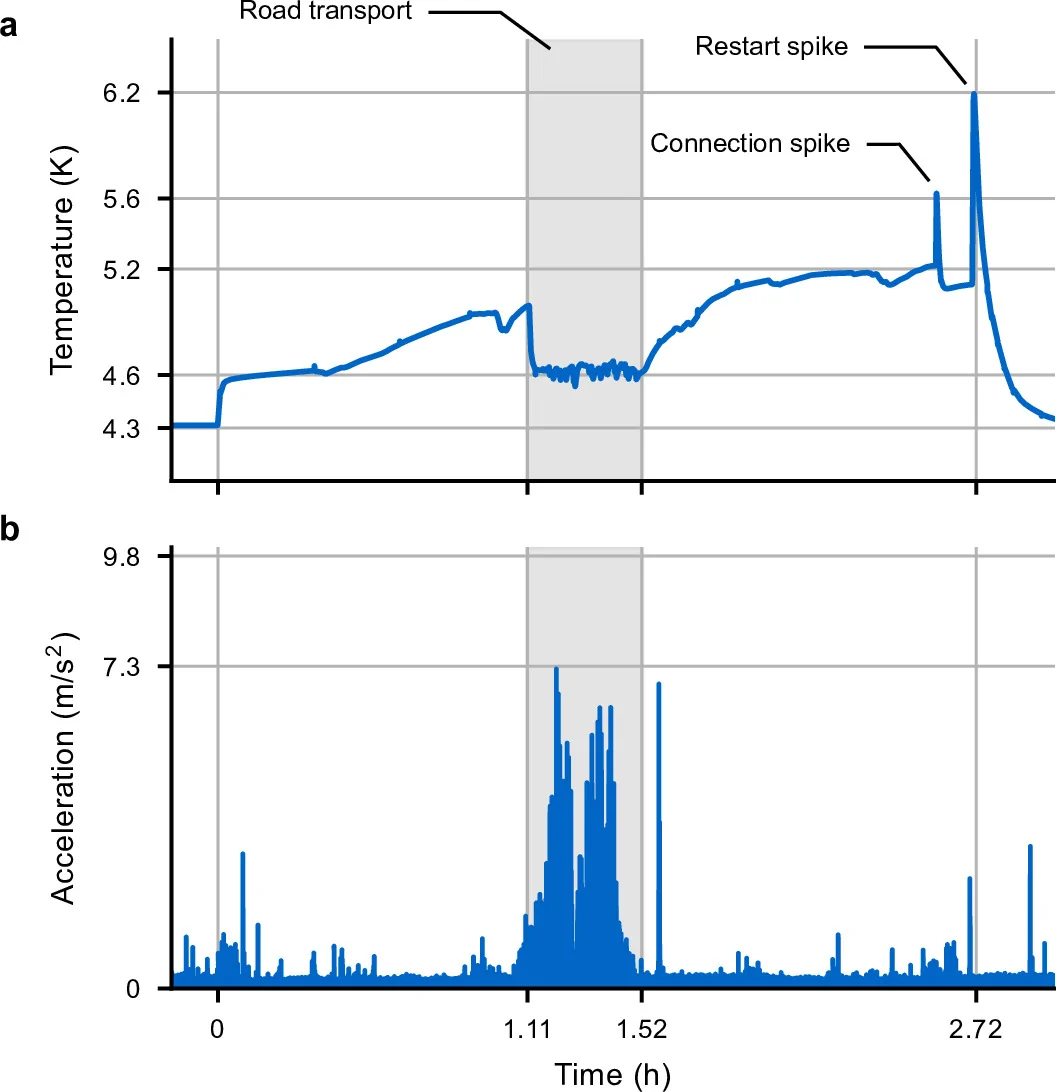
Transport sensor data. **a**, Magnet temperature and **b**, total acceleration measured during autonomous operation. The zero on the horizontal axis marks the shutdown time of the cryocooler. The gray bar indicates the time interval corresponding to the road transport.

With the cryocooler switched off, the magnet temperature (Extended Data Fig. 7a) gradually increases. Once the cryocooler is inactive, the pulse tubes act as a thermal conduction path to the magnet, which remains thermally linked to the cryocooler. Consequently, the magnet temperature increases from 4.3 K to an equilibrium value of about 5.2 K. During the 2-h and 43-min transport sequence, about 11 l of LHe helium evaporated, corresponding to nearly half of the initial LHe buffer reservoir.

The overall temperature evolution during antiproton transport was consistent with that observed during the proton transport rehearsal^10^. During road transport (1.11–1.52 h), accelerations of the transport frame (Extended Data Fig. 7b), including vibration-induced peaks, generated turbulence in the LHe buffer tank. This enhanced magnet cooling and maintained the temperature near 4.6 K throughout truck transport. Transport was performed using a medium-duty truck equipped with air suspension and an automatic transmission. To reduce mechanical shocks and high-frequency vibrations, a 5-t steel plate was installed in the truck bed to soften the effective suspension response during transport.

Transient temperature spikes were observed at 2.57 and 2.72 h of autonomous operation. The first spike occurred during connection of the cryocooler flexlines. During disconnection, a pressure differential developed between the flexlines and the transport-frame-mounted cooling system, with lower pressure in the flexlines. On reconnection, the helium pressure equilibrated, transiently heating the 4 K stage and causing a temporary increase in magnet temperature. The second spike occurred on cryocooler restart at the end of transport. During inactivity, helium gas within the cryocooler warmed, such that initial compressor operation transferred heat into the 4 K stage before steady-state cooling resumed. Following restart of the cooler, the magnet temperature returned to its initial operating temperature.

Roughly 50% of the UPS battery capacity was consumed during transport. During stationary periods, the UPS was temporarily reconnected to external power to preserve battery capacity. Vacuum conditions remained stable throughout transport. Pressure spikes reaching the 10^−7^-mbar level were observed in the cryostat vacuum chamber and attributed to gas release from the multilayer insulation surrounding the thermal stages of the magnet. In contrast, the inlet-chamber pressure remained below the gauge detection threshold of 1 × 10^−9^ mbar, with only occasional vibration-induced outgassing spikes below 3 × 10^−9^ mbar.

## Online content

Any methods, additional references, Nature Portfolio reporting summaries, source data, extended data, supplementary information, acknowledgements, peer review information; details of author contributions and competing interests; and statements of data and code availability are available at https://doi.org/10.1038/s41586-026-11019-z.

## Supplementary information

Peer Review File

## Source data

Source Data Fig. 2

Source Data Fig. 3

Source Data Fig. 4

## Figures and Tables

**Extended Data Table 1:**
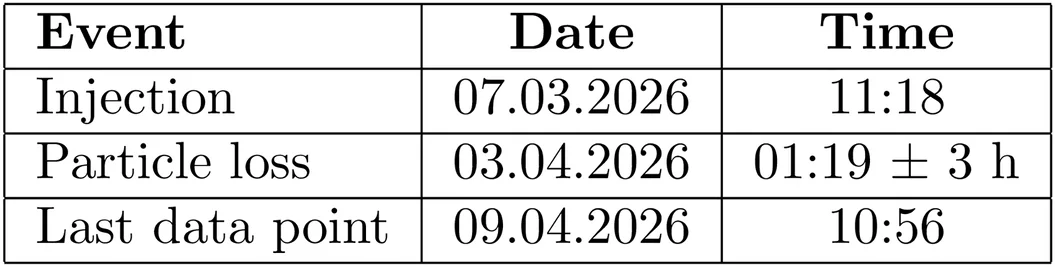
Antiproton reservoir event timeline

Key reservoir events with corresponding dates and times. The uncertainty in the particle-loss time is given in the time column. Times are reported in 24-hour format.

**Extended Data Table 2:**
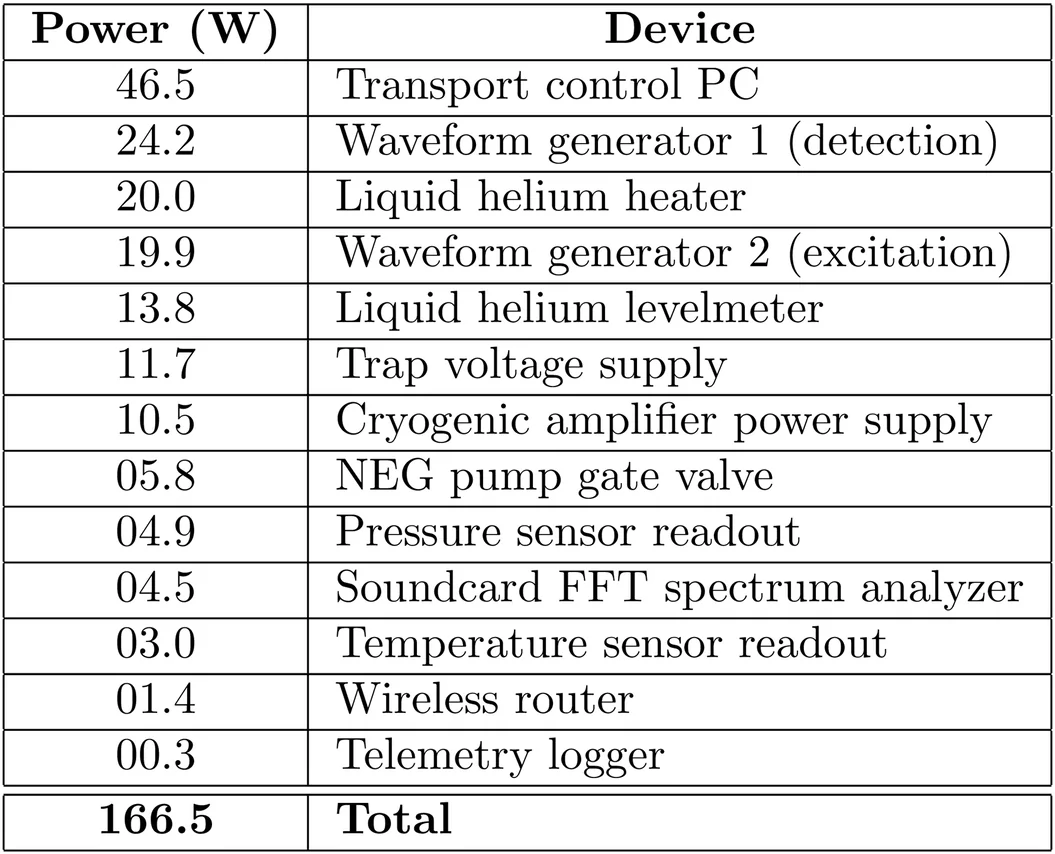
Power consumption

The average power consumption in transport configuration broken down by devices from highest consumption to lowest.

## Data Availability

Source data are provided with this paper. The remaining datasets will be made available by the corresponding author or by S.U. (stefan.ulmer@cern.ch) on request.

## Code availability

Analysis codes will be made available by the corresponding author or S.U. (Stefan.Ulmer@cern.ch) on request.
