# Supplementary material for: Road transport of trapped antiprotons
Source: Nature. 2026 Sep 16;657(8133):906–11. doi: 10.1038/s41586-026-11019-z (PMC13600938; doi:10.1038/s41586-026-11019-z)
Supplement: Supplementary file 1 [file 41586_2026_11019_MOESM1_ESM.pdf]

nature portfolio

Peer Review File

Road Transport of Trapped Antiprotons

Corresponding Author: Dr Christian Smorra

Any redactions in this file are there to maintain patient confidentiality, the confidentiality of unpublished data, or to remove third-party material.

This file contains all reviewer reports in order by version, followed by all author rebuttals in order by version.

Version 0:

Reviewer comments:

Referee #1

(Remarks to the Author)
This is an excellent manuscript and I would like to congratulate the authors to the result.

A) Summary of the key results
This manuscript reports the first transport of antiprotons in the BASE-STEP transportable Penning trap apparatus. After the previous paper [10] which reported the first transport of protons and described the Penning trap, magnet and auxiliary systems, this manuscript gives a an overview of additional improvements and procedures that were developed. Compared to [10], the presented work demonstrates exceptional control of the trap, ultra-high vacuum and cooling and monitoring techniques, without which a loss-less transport of antiprotons would not have been possible. Furthermore, it shows the ability to extract part of the antiproton reservoir down to single particles, the last step in the process necessary for offline measurements of antiproton properties.

B) Originality and significance: if not novel, please include reference
The BASE collaboration is currently the only one that has demonstrated sufficient control of its apparatus and achieved a vacuum that is sufficient to catch, store and transporting antiprotons to another facility.

C) Data & methodology: validity of approach, quality of data, quality of presentation
The data and methodology are well described and appropriate, quality of data and presentation are very high

D) Appropriate use of statistics and treatment of uncertainties
Authors use appropriate statistical methods

E) Conclusions: robustness, validity, reliability
The conclusions are robust and represent well what is presented in the main text and methods

F) Suggested improvements: experiments, data for possible revision
No comments about data or experiments, I have few comments about figures in the main text:
- Fig. 1a - From the color legend, it looks like Catching electrods are C01-C03 but also the left-most, C15 (?). However, from Methods I understood that the catching potential is only in C01-03. If that's the case, keeping C15 white in this schema might avoid confusion.
- Fig.1b - I understand that for clarity purposes you chose to present only the parts used in this parts, however a curious reader might wonder what is the middle section of your trap stack (I guess the storage trap?). You could consider adding it, though it might introduce confusion between catching and storage electrodes in CT and then ST.
- Fig. 2 - The capacity of UPS is maybe unnecessary here, both this and the BASE-STEP power consumption values are in Methods

G) References: appropriate credit to previous work?
Authors use appropriate references to previous work. There might be small changes of editorial character to make the flow of text smoother, eg. not repeating reference to CPT invariabce [2] at the beginning of the main text as it was already mentioned in the abstract.

8) Clarity and context: lucidity of abstract/summary, appropriateness of abstract, introduction and conclusions
The paper is clear, well structured and very well written.

Referee #2

(Remarks to the Author)
Summary

In this article, the authors report a milestone achievement: the first transport of antiprotons away from the CERN Antimatter Factory (AMF) using a high-precision, ultra-high-vacuum Penning trap. This represents a crucial step for the BASE collaboration's program of performing the highest-precision measurements of the antiproton's charge-to-mass ratio and magnetic moment for stringent tests of CPT invariance. More broadly, however, it marks the first step toward making antiprotons accessible beyond accelerator-based experiments at CERN, paving the way for a wider deployment of antimatter in precision physics.

The manuscript first reviews the BASE collaboration's previous on-site measurements and explains how their precision is currently limited by the magnetic field environment in the AMF experimental hall. The authors then present an overview of the transportable Penning trap system, BASE-STEP, which is intended to enable the transport of small numbers of antiprotons from the AMF to laboratories with significantly lower magnetic noise. The authors describe their antiproton loading procedure, which typically captures approximately 90 antiprotons in the BASE-STEP trap, and report the successful demonstration of transporting 92 antiprotons over a distance of 3.5 km by truck around the CERN Meyrin site under normal traffic conditions. The transport was monitored continuously and was demonstrated to be lossless. This impressive demonstration establishes the feasibility of transporting antiprotons while maintaining confinement and is expected to enable an estimated two-order-of-magnitude improvement in the precision of future BASE measurements. More importantly, it opens the door to precision antimatter experiments outside the AMF. Given that antimatter has historically been confined to accelerator facilities, this work represents a transformative advance that could enable high-precision antimatter research to be more broadly accessible within the scientific community.

Major Comments:

The reviewers have no major comments and have identified no flaws that should prevent this article's publication.

Minor Comments:

- In Figure 3 the red bars indicate “transport preparation tests”, but it is not clear exactly what this consisted of. Running autonomously on the UPSs as mentioned at the top of Page 4? Craning the frame? A bit more description of this would be helpful to compare these red flagged regions with the transport part in Figure 3.

- On page 4 the detector noise spectra are discussed. The text mentions that ”during the first round” there was a frequency shift of 122 Hz, visible in what looks like a 30 min time window between Figures 2c and 2d. But what does “first round” mean here?

- Figure 3 is a key plot because it demonstrates the lossless transport. However, if we look at the region of this figure after the transport up until about day 25, it looks naively that there are many more large excursions around the mean than there were before transport, for example comparing post transport with the period of days 7-14. One could assume that it was manipulations trying to correct this that led to the antiproton loss around day 28. Can you comment on what the origin of these excursions are? Is it something that was induced during transport or just bad luck with external conditions in the AMF ?

- When the effect of temperature fluctuations is discussed in the main text, it may be useful to reference explicitly the details of this analysis in the Methods section, Extended Data Fig. F1.

-Again on page 4, there is a sentence that reads “Although limited data quality during transport allowed extraction of only a few dipwidth points, the antiprotons have been continuously tracked". But then you also discuss detector noise spectra at 1 minute intervals above, and it is not clear how long an acquisition takes. Can this explanation be cleaned up slightly to describe exactly what was acquired during transport, how long, at what intervals?

- In the Methods section B, the particle count calibration process is described. This includes a measurement at 993 mT (catching field) and 136 mT (transport field) and a reference to a 880mT field that is only seen in Fig. 3 and without discussion. A comment from the authors about this field setting in the text would be clarifying and an explicit measurement of the calibration curve would be an improvement over the indirect method cited in the text. Alternatively, an exclusion of the data and how it is calibrated would be acceptable.

- In the Methods section B where the application of magnetron side-band cooling is described, reference to the proton transport test (citation 10) is made where the authors comment that for the proton test, the width expansion could not be seen on the time scale of 4 hours. It is unclear from where the “higher torque” present in this test emerges; for example, if there are differences in the trap configuration that give rise to this change. The cooling process is otherwise well motivated and documented and the direct estimation of uncertainty in the particle number strengthens the argument of the paper.

- In the Methods section B (page 9 left), both Figures B1.b and B2 are lacking (visible) error bars. This warrants a comment from the authors. Further, the inset in Fig. B1.b is not addressed in the text or the caption. The pair of points at particle number = 1, which are both red (from proton data) is confusing. Is this statistical fluctuation from two proton runs or two separate proton runs with different field settings?

- In Methods section D there is a comment “[the new valve] is supposed to have a lower leak rate than the metal-on-metal sealed version”. This comparison feels imprecise and could be strengthened by a specific estimate or measurement.

- In Methods section E “all electronics except room-temperature amplifiers are powered by a dedicated UPS”: were the room-temperature amplifiers powered by their own batteries? This would be standard practice but, as written, it leaves an information gap.

- In the conclusion, prospects of increasing trapping yield to 1000 antiprotons is mentioned with the addition of a rotating wall to allow for greater capacity still. Perhaps it is outside the scope of this paper, but it would be interesting to know; is there a known or expected limit to the trapping capacity (or transport capacity) of a trap with a rotating wall? In view of the “democratization” of antiprotons, it is useful to have order of magnitude ideas of how many antiprotons one could expect to deliver to other labs in a near-ish future.

Referee #3

(Remarks to the Author)
I co-reviewed this manuscript with one of the reviewers who provided the listed reports.

Open Access This Peer Review File is licensed under a Creative Commons Attribution 4.0 International License, which permits use, sharing, adaptation, distribution and reproduction in any medium or format, as long as you give appropriate credit to the original author(s) and the source, provide a link to the Creative Commons license, and indicate if changes were made.
In cases where reviewers are anonymous, credit should be given to 'Anonymous Referee' and the source.
The images or other third party material in this Peer Review File are included in the article’s Creative Commons license, unless indicated otherwise in a credit line to the material. If material is not included in the article’s Creative Commons license and your intended use is not permitted by statutory regulation or exceeds the permitted use, you will need to obtain permission directly from the copyright holder.
To view a copy of this license, visit https://creativecommons.org/licenses/by/4.0/

Referee #1 (Remarks to the Author):

This is an excellent manuscript and I would like to congratulate the authors to the result.

Answer: We would like to thank referee #1 for taking his/her valuable time to review our manuscript and for the positive assessment of our work. We reply to the suggested improvements in detail below.

A) Summary of the key results
This manuscript reports the first transport of antiprotons in the BASE-STEP transportable Penning trap apparatus. After the previous paper [10] which reported the first transport of protons and described the Penning trap, magnet and auxiliary systems, this manuscript gives a an overview of additional improvements and procedures that were developed. Compared to [10], the presented work demonstrates exceptional control of the trap, ultra-high vacuum and cooling and monitoring techniques, without which a loss-less transport of antiprotons would not have been possible. Furthermore, it shows the ability to extract part of the antiproton reservoir down to single particles, the last step in the process necessary for offline measurements of antiproton properties.

B) Originality and significance: if not novel, please include reference
The BASE collaboration is currently the only one that has demonstrated sufficient control of its apparatus and achieved a vacuum that is sufficient to catch, store and transporting antiprotons to another facility.

[...]

F) Suggested improvements: experiments, data for possible revision

No comments about data or experiments, I have few comments about figures in the main text:
- Fig. 1a - From the color legend, it looks like Catching electrods are C01-C03 but also the left-most, C15 (?). However, from Methods I understood that the catching potential is only in C01-03. If that's the case, keeping C15 white in this schema might avoid confusion.

Answer: Thank you for pointing this out – we modified the figure accordingly.

- Fig.1b - I understand that for clarity purposes you chose to present only the parts used in this parts, however a curious reader might wonder what is the middle section of your trap stack (I guess the storage trap?). You could consider adding it, though it might introduce confusion between catching and storage electrodes in CT and then ST.

Answer: We omitted the storage trap since it was not used in the reported findings. Following this remark, we decided to put the storage trap label back into Fig 1b and modified the caption.

- Fig. 2 - The capacity of UPS is maybe unnecessary here, both this and the BASE-STEP power consumption values are in Methods

Answer: We removed UPS capacity as suggested.

G) References: appropriate credit to previous work?
Authors use appropriate references to previous work. There might be small changes of editorial character to make the flow of text smoother, eg. not repeating reference to CPT invariabce [2] at the beginning of the main text as it was already mentioned in the abstract.

Answer: We removed ref. [2] at the beginning of the main text.

8) Clarity and context: lucidity of abstract/summary, appropriateness of abstract, introduction and conclusions
The paper is clear, well structured and very well written.

Answer: We would like to thank referee #1 once more for reviewing our manuscript. We assert that all statements made by referee #1 show that our manuscript fulfils the criteria and support the publication of our manuscript. We have also addressed and implemented all requested changes.

Referee #2 (Remarks to the Author):

Summary

In this article, the authors report a milestone achievement: the first transport of antiprotons away from the CERN Antimatter Factory (AMF) using a high-precision, ultra-high-vacuum Penning trap. This represents a crucial step for the BASE collaboration's program of performing the highest-precision measurements of the antiproton's charge-to-mass ratio and magnetic moment for stringent tests of CPT invariance. More broadly, however, it marks the first step toward making antiprotons accessible beyond accelerator-based experiments at CERN, paving the way for a wider deployment of antimatter in precision physics.

The manuscript first reviews the BASE collaboration's previous on-site measurements and explains how their precision is currently limited by the magnetic field environment in the AMF experimental hall. The authors then present an overview of the transportable Penning trap system, BASE-STEP, which is intended to enable the transport of small numbers of antiprotons from the AMF to laboratories with significantly lower magnetic noise. The authors describe their antiproton loading procedure, which typically captures approximately 90 antiprotons in the BASE-STEP trap, and report the successful demonstration of transporting 92 antiprotons over a distance of 3.5 km by truck around the CERN Meyrin site under normal traffic conditions. The transport was monitored continuously and was demonstrated to be lossless. This impressive demonstration establishes the feasibility of transporting antiprotons while maintaining confinement and is expected to enable an estimated two-order-of-magnitude improvement in the precision of future BASE measurements. More importantly, it opens the door to precision antimatter experiments outside the AMF. Given that antimatter has historically been confined to accelerator facilities, this work represents a transformative advance that could enable high-precision antimatter research to be more broadly accessible within the scientific community.

Major Comments:

The reviewers have no major comments and have identified no flaws that should prevent this article's publication.

Answer: We would like to thank referee #2 very much for the evaluation of our manuscript and are delighted about the review and the conclusions. We assert that referee #2 also supports the publication of our manuscript.

Minor Comments:

- In Figure 3 the red bars indicate "transport preparation tests", but it is not clear exactly what this consisted of. Running autonomously on the UPSs as mentioned at the top of Page 4? Craning the frame? A bit more description of this would be helpful to compare these red flagged regions with the transport part in Figure 3.

Answer: We thank the reviewer for this suggestion. We agree that the original figure caption did not sufficiently explain the activities represented by the red highlighted regions. We have therefore expanded the caption of Fig. 3 to clarify that the transport preparation tests comprised two stationary autonomous-operation tests and one overhead crane transport performed prior to the road transport. This makes it easier to relate the highlighted transport preparation periods to the subsequent road transport shown in the figure.

- On page 4 the detector noise spectra are discussed. The text mentions that "during the first round" there was a frequency shift of 122 Hz, visible in what looks like a 30 min time window between Figures 2c and 2d. But what does "first round" mean here?

Answer: We thank the reviewer for pointing out this ambiguity. We have clarified the text by replacing "during the first round" with "during the first of the two laps" to explicitly indicate that this refers to the first lap around the Meyrin site.

- Figure 3 is a key plot because it demonstrates the lossless transport. However, if we look at the region of this figure after the transport up until about day 25, it looks naively that there are many more large excursions around the mean than there were before transport, for example comparing post transport with the period of days 7-14. One could assume that it was manipulations trying to correct this that led to the antiproton loss around day 28. Can you comment on what the origin of these excursions are? Is it something that was induced during transport or just bad luck with external conditions in the AMF?

Answer: We thank the reviewer for this observation. We had also noticed these apparent excursions during the data-taking period and investigated their origin at the time. We found that they are not induced by the transport or the trap system itself but are correlated with external noise from the AMF while neighbouring systems were being commissioned in March 2026. During this period, a noise peak intermittently drifted onto the resonator spectrum. Whenever this occurred, the resonance dip was partially distorted, causing the fitting routine to underestimate the dip width and resulting in the observed downward excursions. The antiproton loss around day 28 is reported on in detail in the main text and methods section. It is not related to these dip-width excursions and was not caused by attempts to compensate for them.

- When the effect of temperature fluctuations is discussed in the main text, it may be useful to reference explicitly the details of this analysis in the Methods section, Extended Data Fig. F1.

Answer: We thank the reviewer for this suggestion. We have added an explicit reference to the Methods section and Extended Data Fig. F1 in the main text, where the magnet temperature evolution and acceleration measurements during transport are discussed.

-Again on page 4, there is a sentence that reads "Although limited data quality during transport allowed extraction of only a few dipwidth points, the antiprotons have been continuously tracked". But then you also discuss detector noise spectra at 1 minute intervals above, and it is not clear how long an acquisition takes. Can this explanation be cleaned up slightly to describe exactly what was acquired during transport, how long, at what intervals?

Answer: We thank the reviewer for pointing out this potential ambiguity. We clarify that the detector noise spectra were acquired continuously, without interruption, at 1 minute intervals throughout the entire transport. The spectra shown in Fig. 2c–e are examples of the recorded data. From these spectra, the axial frequency and dip width were extracted; however, only spectra with sufficient signal-to-noise ratio yielded reliable dip-width values. We have therefore clarified the text by modifying the sentence to state that "limited data quality during transport allowed extraction of only a few reliable dip-width points from the recorded spectra," distinguishing between the continuous acquisition of spectra and the subset suitable for dip-width extraction.

- In the Methods section B, the particle count calibration process is described. This includes a measurement at 993 mT (catching field) and 136 mT (transport field) and a reference to a 880mT field that is only seen in Fig. 3 and without discussion. A comment from the authors about this field setting in the text would be clarifying and an explicit measurement of the calibration curve would be an improvement over the indirect method cited in the text. Alternatively, an exclusion of the data and how it is calibrated would be acceptable.

Answer: We thank the referee for this comment. To avoid confusion, we followed your suggestion and added a brief sentence in the Methods section explaining the purpose of this intermediate field. We decided however to keep the data and field strength in the manuscript to show the continuous monitoring of the trapped antiprotons, and prefer this rather than excluding the data and explaining the gap in the data acquisition.

The intermediate field of 880 mT was reached in order to tune the trapped antiprotons to a cyclotron frequency detector. However, this step is not relevant to the discussion of the experiment and was therefore omitted from the main text. The field value nevertheless appears in Fig. 3 because it was part of the actual magnetic-field sequence.

Regarding the calibration, the indirect determination of the single-particle dip width at 880 mT is sufficiently accurate for the purposes of this work. The detector parallel resistance, R_p, is measured directly at all three field values of 136 mT, 880 mT and 993 mT. Using the established proportionality $\Delta\nu_{z,1} \propto R_p$ together with the direct calibrations at 993 mT and 136 mT therefore provides a reliable estimate of the dip width per particle. Moreover, the particle number is independently constrained by the directly calibrated measurements immediately before and after the 880 mT setting, both of which yield 92 trapped antiprotons, which shows that the particle number assignment at 880 mT is correct. We also assert that the conclusions on the preservation of antiprotons is not affected by the intermediate measurement at 880 mT.

- In the Methods section B where the application of magnetron side-band cooling is described, reference to the proton transport test (citation 10) is made where the authors comment that for the proton test, the width expansion could not be seen on the time scale of 4 hours. It is unclear from where the "higher torque" present in this test emerges; for example, if there are differences in the trap configuration that give rise to this change. The cooling process is otherwise well motivated and documented and the direct estimation of uncertainty in the particle number strengthens the argument of the paper.

Answer: We thank the reviewer for pointing out that the origin of the increased torque was not sufficiently addressed in the original manuscript. The manuscript has been slightly revised accordingly to emphasize that the torque-driven radial expansion is an observed effect that is monitored and compensated for but that the origin is not identified.

Potential reasons for the torque are a change of the trap angle due to modifications on the cryogenic support structure, or a breaking of the azimuthal symmetry due to patch potentials (potentially caused by the antiproton injection) or a tiny current leak on the radially segmented electrode. It is our interest to restore the previous performance and decrease the radial expansion rate. We will review this issue when the trap system will be opened after the current run (in October 2026) and include also rotating-wall electrodes that can actively counteract these torques and prevent radial expansion of even larger

antiproton clouds.

- In the Methods section B (page 9 left), both Figures B1.b and B2 are lacking (visible) error bars. This warrants a comment from the authors. Further, the inset in Fig. B1.b is not addressed in the text or the caption. The pair of points at particle number = 1, which are both red (from proton data) is confusing. Is this statistical fluctuation from two proton runs or two separate proton runs with different field settings?

Answer: We thank the reviewer for pointing out the ambiguity caused by the original labelling of Fig. B1b.

The two points at (N=1) are both proton measurements recorded at different magnetic-field strengths: one at \SI{136}{\milli\tesla} and one at \SI{993}{\milli\tesla}, where the latter was obtained after ramping the magnet following the reduction to a single particle.

In the original figure, the color coding by particle species made these two points appear as if they represented two proton measurements under the same calibration conditions, which was misleading. Since the relevant parameter for the dip-width calibration is the magnetic field strength, rather than the particle species (as proton and antiproton dip widths are identical), we have modified the figure labelling to distinguish the data sets according to magnetic field. In the revised figure, the two (N=1) points correspond naturally to the single-particle reference points for the two independent calibration lines.

We have also clarified the presentation of Fig. B1b by adding an explanation of the inset in the figure caption and the corresponding text in the Methods section. The inset now highlights the low-particle-number region (N=1-3), where deviations from the linear calibration would have the most apparent impact on particle-number assignment. Furthermore, we have added a note that the statistical uncertainties of the measured dip widths are smaller than the marker size (order 100 mHz) and were therefore omitted from the figure for clarity.

In Fig B2 we have omitted the statistical uncertainty of the individual data points since the measurement uncertainty should rather be inferred from the distribution of the data points. We quote now the RMS fluctuation of the dip width shift in the figure caption to give an explicit number for the measurement uncertainty.

- In Methods section D there is a comment "[the new valve] is supposed to have a lower leak rate than the metal-on-metal sealed version". This comparison feels imprecise and could be strengthened by a specific estimate or measurement.

Answer: The leak rate of the inlet valve needs to be determined under cryogenic conditions, which requires the installation and cooldown in a dedicated cryostat test setup, and this was not yet possible due to a lack of time. It was necessary to install it immediately into the trap system to be able to start up for the antiproton beam taking of the ongoing physics run in 2025. There was no opportunity to remove this essential component of our trap system yet. We are currently replicating the valve and are preparing a test setup to characterize the leak rates in cryogenic conditions, but these are ongoing investigations and beyond scope of this paper. We rephrased this part to be clear on the status of the lacking test measurement of the device.

- In Methods section E "all electronics except room-temperature amplifiers are powered by a

dedicated UPS": were the room-temperature amplifiers powered by their own batteries? This would be standard practice but, as written, it leaves an information gap.

Answer: We thank the reviewer for pointing out this missing information. The room-temperature amplifiers are powered by their own batteries, as shown in Fig. E1. We have now clarified this in the Methods section including a reference to the figure.

- In the conclusion, prospects of increasing trapping yield to 1000 antiprotons is mentioned with the addition of a rotating wall to allow for greater capacity still. Perhaps it is outside the scope of this paper, but it would be interesting to know; is there a known or expected limit to the trapping capacity (or transport capacity) of a trap with a rotating wall? In view of the "democratization" of antiprotons, it is useful to have order of magnitude ideas of how many antiprotons one could expect to deliver to other labs in a near-ish future.

Answer:

The limits to the trapping capacity of our specific trap system equipped with a rotating wall are indeed beyond the scope of this work. A systematic study of these limits will only become possible once the planned upgrade has been implemented. At present, the quoted value of 1000 antiprotons is a conservative target that we are confident can be achieved with significant margin for further optimization.

As a reference, the current state of the art in Penning-Malmberg traps with rotating wall compression is, to our knowledge, the recent result from the GBAR collaboration, which reported the storage of 6×10^7 antiprotons by accumulating 18 injections (B. Lee *et al.*, *Record accumulation of antiprotons in a Penning-Malmberg Trap and their preparation for improved production of antihydrogen beams*, *Journal of Physics G: Nuclear and Particle Physics*, 2026).

The Brillouin limit provides a principle upper limit to the density of a non-neutral plasma with n = $B^2/(2 \mu_0 mc^2)$, e.g. 3 10^9 antiprotons per cm^3 for 1 T field. Such large antiproton numbers require therefore in comparison to BASE-STEP larger magnetic fields (>1 T) and larger traps (d > 12 mm). We would like to emphasize, however, that the BASE-STEP concept is primarily intended to supply antiprotons for non-destructive precision measurements, for which substantially fewer particles are both sufficient and preferable. Consequently, our goal is not to maximize the number of stored antiprotons for its own sake, but rather to provide the particle numbers required by the intended applications.

For transport applications with larger particle numbers, the PUMA experiment is currently developing a transportable Penning-Malmberg trap with a design concept targeting the storage and transport of approximately 10^9 antiprotons. This likely represents the order of magnitude that may become feasible for delivery to external laboratories in the foreseeable future.

For the "democratization" of antiprotons it is primarily necessary to have more transport trap systems available, as BASE-STEP and PUMA both have an experiment program for the next decade. We are currently working on transportable permanent magnet traps that will operate at lower field (280 mT – 500 mT) and small volumes therefore not at high-end particle numbers, but that can supply antiprotons for non-destructive experiments with lower effort than with the present system.

Referee #3 (Remarks to the Author):

I co-reviewed this manuscript with one of the reviewers who provided the listed reports.

Answer: We would like to thank also referee #3 for taking his/her time to review our manuscript, and for the joined preparation of the listed report. Thank you very much!
